# On prediction of aided behavioural measures using speech auditory brainstem responses and decision trees

**DOI:** 10.1371/journal.pone.0260090

**Published:** 2021-11-16

**Authors:** Emanuele Perugia, Ghada BinKhamis, Josef Schlittenlacher, Karolina Kluk

**Affiliations:** 1 Manchester Centre for Audiology and Deafness, School of Health Sciences, Faculty of Biology, Medicine and Health, University of Manchester, Manchester Academic Health Science Centre, Manchester, United Kingdom; 2 Department of Communication and Swallowing Disorders, Rehabilitation Hospital, King Fahad Medical City, Riyadh, Saudi Arabia; Universidade Estadual de Ciencias da Saude de Alagoas, BRAZIL

## Abstract

Current clinical strategies to assess benefits from hearing aids (HAs) are based on self-reported questionnaires and speech-in-noise (SIN) tests; which require behavioural cooperation. Instead, objective measures based on Auditory Brainstem Responses (ABRs) to speech stimuli would not require the individuals’ cooperation. Here, we re-analysed an existing dataset to predict behavioural measures with speech-ABRs using regression trees. Ninety-two HA users completed a self-reported questionnaire (SSQ-Speech) and performed two aided SIN tests: sentences in noise (BKB-SIN) and vowel-consonant-vowels (VCV) in noise. Speech-ABRs were evoked by a 40 ms [da] and recorded in 2x2 conditions: aided vs. unaided and quiet vs. background noise. For each recording condition, two sets of features were extracted: 1) amplitudes and latencies of speech-ABR peaks, 2) amplitudes and latencies of speech-ABR F0 encoding. Two regression trees were fitted for each of the three behavioural measures with either feature set and age, digit-span forward and backward, and pure tone average (PTA) as possible predictors. The PTA was the only predictor in the SSQ-Speech trees. In the BKB-SIN trees, performance was predicted by the aided latency of peak F in quiet for participants with PTAs between 43 and 61 dB HL. In the VCV trees, performance was predicted by the aided F0 encoding latency and the aided amplitude of peak VA in quiet for participants with PTAs ≤ 47 dB HL. These findings indicate that PTA was more informative than any speech-ABR measure, as these were relevant only for a subset of the participants. Therefore, speech-ABRs evoked by a 40 ms [da] are not a clinical predictor of behavioural measures in HA users.

## Introduction

One of the overarching goals of research in the audiology/hearing sciences is to develop objective measures to assess hearing aid (HA) outcome and benefits. Typically, a person’s benefit from HAs is based on their ability to perceive speech. This is evaluated with self-report questionnaires (e.g., [1]), or with performance on speech-in-noise (SIN) tests, such as Bamford-Kowal-Bench sentences in noise (BKB-SIN) and vowel-consonant-vowels (VCV) in noise [2, 3]. Since the clinical assessment of HA outcomes for some individuals with disabilities cannot be performed using these behavioural measures, there is a need to develop objective measures for such populations. Possible candidates could be based on Auditory Brainstem Responses (ABRs). Indeed, ABR testing is already a clinical routine to estimate hearing thresholds [4, 5]. ABRs are electrophysiological event-related potentials, time-locked to a transient stimulus, and generated by the brainstem auditory pathways [5]. ABRs can also be evoked in response to different types of sounds, including short consonant-vowel (CV) speech token stimuli and, in this case, they are termed speech-ABRs [6–8].

The CVs [ba] [da] and [ga] are typical stimuli used to evoke speech-ABRs [7, 8]. The duration of the stimuli may vary between shorter (e.g. 40 ms) and longer (e.g. 170 ms) [7, 8]. The morphology of speech-ABRs to CV stimuli is characterised by an onset, transition and a sustained (frequency-following) responses [8, 9]. The onset is evoked by the consonant onset, the transition is evoked by the transition from consonant to vowel and the frequency-following response (FFR) is mainly synchronised to the fundamental frequency (F0) of the vowel [8, 9]. The term Envelope Following Response (EFR) may also be used to remark the variation of the responses in frequency [10]. Feature extraction from speech-ABRs can be performed in the time domain by way of a series of peaks, or in the frequency domain estimating F0 and successive harmonics [8, 11]. The stimulus duration (of the same stimuli, i.e. the same CV), like 50 or 170 ms, does not have significant effects on amplitudes and latencies of speech-ABR peaks, but the number of epochs required for detection increases as stimulus duration increases [7]. A longer stimulus combined with a larger number of epochs results in longer speech-ABR recording times [7], hence longer stimuli are less clinically applicable than shorter stimuli.

Anderson & Kraus [11] suggested assessing hearing-in-noise difficulties and HA fitting outcomes in older adults via speech-ABRs. Despite speech-ABRs being promising objective measures of speech perception, few studies were devoted to investigating speech-ABRs in adults with hearing loss or with HAs, and their relation to SIN performance. For instance, Easwar et al. [12] measured aided speech discrimination scores and sound quality ratings as well as aided EFRs to a speech token [susa∫i] as a function of the stimulus bandwidth in 20 adults with HAs. The two behavioural measures increased with increase in bandwidth and were positively correlated with both the EFR amplitudes and the number of EFRs detected. In order to evaluate acclimatization and benefit from HAs, Karawani et al. [13] compared the scores on subscales of a self-report questionnaire (i.e. Abbreviated Profile of Hearing Aid Benefit, or APHAB) with FFRs evoked by a 170-ms speech syllable /ga/, both were assessed twice at six months apart. The differential measures across time revealed that F0 amplitude decreased as aversion to sound decreased (i.e. on the aversiveness APHAB subscale), and peak latencies decreased as ease of communication increased. However, Karawani et al. [13] collected further data (e.g. performance on a SIN tests, data from a control group) that were not used against FFR measures. In addition, both Easwar et al. [12] and Karawani et al. [13] explored relationships between speech-ABRs and behavioural and self-report measures via correlations, as such it remains unclear whether speech-ABRs could predict behavioural measures.

The prediction of behavioural measures with speech-ABRs was attempted recently by BinKhamis et al. [6]. In their second experiment, adults with sensorineural hearing loss (SNHL) performed the BKB-SIN and VCV in noise tests, and self-reported speech understanding while wearing a HA. Moreover, speech-ABRs evoked by the 40 ms [da] were recorded in 2x2 conditions: aided vs. unaided and quiet vs. background noise. Importantly, the same HA was fitted for all participants to ensure consistency. Using linear regression models, BinKhamis et al. [6] found that the two aided behavioural measures and the self-report measure were predicted only by participants’ pure tone average (PTA: average thresholds at 500, 1000, 2000, and 4000 Hz) hearing thresholds (as the performance worsened with decreasing PTAs), and aided speech-ABRs were not significant predictors of behavioural and self-report measurements. It was suggested that F0 encoding latencies and amplitudes (which were the speech-ABR features entered in the regression models) and behavioural SIN tests are measuring different auditory processes. The choice of using speech-ABR F0 encoding instead of the classical peaks had two reasons: 1) F0 is a key feature for speech understanding in noise; 2) F0 encoding had fewer missing data points than speech-ABR peaks, which in turn led to fewer participants being excluded from the analyses (only 11 participants were excluded from the regression analyses when using F0 encoding—i.e. a total of 81 participants included, as opposed to 39 participants who would have been excluded if speech-ABR peaks were used in the regression analyses, i.e. only 53 participants would have been included).

In the current study, we used the data from BinKhamis et al. [6] to predict behavioural measures and self-report with speech-ABRs using regression trees. To the best of our knowledge, this is the largest dataset containing a combination of speech-ABRs, behavioural, and self-report measures in adults with SNHL. The participants were also representative of audiology clinical populations as they had a wide range of PTAs. In order to predict a response variable, the regression tree recursively partitions the predictors space into non-overlapping rectangles minimising the mean squared error, such that the rectangles are increasingly homogeneous relative to the response variable [14, 15]. Regression trees may outperform the linear regression modelling used by BinKhamis et al. [6] in three ways. First, trees lead to a better understanding of the predictive structure of the data, disclosing plausible non-linear boundaries. For instance, when subtracting EEG waves of congruent sentences from incongruent sentences recorded at different signal-to-noise ratios (SNRs) in adults with normal hearing, Jamison et al. [16] observed that the N400 amplitude (i.e. about 400 ms from word onset) decreased from 4 to 1 dB SNR but increased at 0 dB SNR, but a linear model would not be able to capture this non-linear pattern. Second, since trees’ terminal rectangles are strongly homogeneous, different sub-groups of participants may be associated with specific speech-ABR features. Third, trees are robust with respect to missing data. Here, the missing data were within speech-ABR peaks and F0 encoding. If a peak is not clearly observable or F0 encoding is not significant, their amplitudes and latencies are missing. Thus, our type of missingness is defined as missing not at random (MNAR) because the probability that an observation (e.g. speech-ABR peak amplitude) is missing is a function of both the value of the observation and other variables (e.g. background noise) in the design [17, 18], as well as the presence/detection of peaks may also be influenced by the degree of hearing loss. The MNAR scenario might be an opportunity because the missing pattern could be predicted by a model [17, 19]. Furthermore, it is plausible to assume that any new data collection, using the same procedure and same participant inclusion criteria and same range of hearing losses, will have a similar proportion of missing data relative to the current one. The treatment of missing data in the decision trees is based on surrogate splits and variables [14]. In a given node, the split of the variable with missing data is predicted using the other non-missing and independent variables with highest association to the split [14, 20]. Hence, highly correlated variables would lead to a better prediction on the surrogate split [14]. Regression trees are not exempt from limitations [15, 21]. The main one is their high variance: a change in the dataset can lead to different series of splits and, hence, tree [15, 21]. Although this is somewhat true with any machine learning algorithms with insufficient data, averages or aggregations of many trees may help to solve this at the expense of the interpretation of the model [15]. One strong reason for using regression trees was to have easy interpretable models. Furthermore, here we re-analysed probably the largest available dataset with both speech-ABR and behavioural measures in HA users. The second limitation is that the partitioning algorithm is biased in favour of categorical predictors with many levels [21, 22]. However, this issue is more severe in classification than regression trees [23] and it is not a concern here, as we did not have categorical predictors. Another limitation of the regression trees is their lack of modelling additive structure [21]. In the case of a small number of additive effects, they would be in different splits, so the structure can be recognised and, hence, relationships between behavioural and speech-ABR measures can be explained.

The aim of this study was to explore, within a non-linear approach, if features of the speech-ABR were significant predictors of behavioural measures. Our hypothesis was that regression trees would reveal a strong relationship between speech-ABRs and behavioural measures, at least for sub-groups of participants given that regression trees are more sensitive than linear models. Further, it was expected that some relationships would be non-linear.

## Materials and methods

### Participants

**Fig 1:**
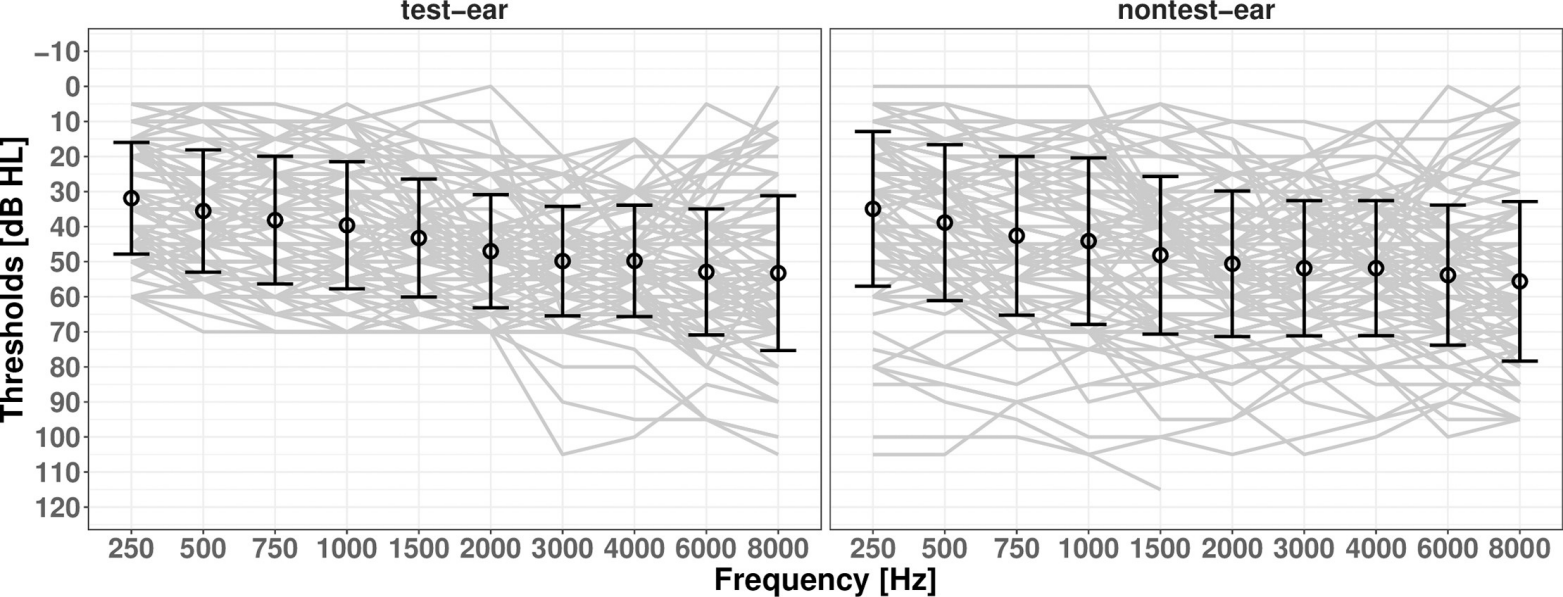
Test-ear (left panel, partially reproduced from [6]) and nontest-ear (right panel) mean (±1 SD) pure tone thresholds (black) with pure tone thresholds for each of the 92 participants (grey).

The participants included in [6] were the same in the current study. The participants were 92 adult HA users (38 men) with SNHL, in the age range 18 to 60 years (distribution of age is shown in Fig 1 in S1 File), with mean (SD) of 50.35 (9.07) years and with mean (SD) of 42.96 (13.58) PTA. The included participants had acquired bilateral SNHL not exceeding 70 dB HL at low to mid frequencies (i.e., between 250 and 2000 Hz) in the better ear or in the aided ear; and they used at least one HA for 3 months minimum. The participants had no history of learning difficulties, neurological disorders, or cognitive impairments. The study was approved by the National Health Services Research Ethics Committee, England (IRAS ID: 226216). All participants provided written informed consent.

### Hearing evaluation and HA fitting

#### Hearing assessment

Otoscopy, tympanometry and pure tone audiometry were performed on both ears in each participant. Ears were examined following the BSA recommended procedure [24]; and tympanometry was performed using the Interacoustics Titan device with a 226-Hz probe tone [25]. Air conduction pure tone thresholds were measured at octave and interoctave frequencies from 250 to 8000 Hz (Fig 1). Bone conduction pure tone thresholds were measured at octave frequencies from 500 to 4000 Hz [26].

#### HA fitting

Each participant was fitted to NAL-NL2 targets [27] with one Oticon opn1 miniRITE (Oticon A/S, Copenhagen, Denmark) HA on the better ear, on the aided ear for participants that were monaural hearing aid users, or on the right ear for participants with symmetrical hearing loss that were binaural hearing aid users. Monaural fitting was chosen to avoid the confound of the better-hearing ear driving the response in cases of asymmetrical hearing losses, to evaluate the potential application of speech-ABRs as an outcome measure for individual HA fitting, and to account for test-ear pure tone thresholds in the analyses. Each HA was fit using an appropriate receiver per-participant hearing thresholds and fitting was adjusted and verified to meet NAL-NL2 targets [27] according to the BSA recommendations [28]. More details on the implementations of the HA Fitting and Verification are available in [6].

### Behavioural measures

#### Speech-in-noise tests

All participants performed two speech-in-noise (SIN) tests while wearing the HA. Fifty percent speech recognition thresholds (SRT-50) were obtained for Bamford-Kowal-Bench [2] sentences in noise (BKB-SIN) and vowel-consonant-vowels (VCVs; [3]) in noise. BKB sentences were pre-recorded, spoken by a male speaker and were presented in eight-talker babble. Sentences were fixed at 65 dB-A and the level of the background babble was adapted with the initial SNR set at + 6 dB. The first four sentence lists (16 sentences per list) were used. Participants were instructed to either repeat each sentence, or as many words as they heard, or as many words as they thought they heard. When all keywords were correct, the SNR was decreased by 3 dB, and if any of the keywords were incorrect, the SNR was increased by 3 dB. BKB-SIN SRT-50 was obtained by averaging the SRT-50 from the four lists. VCVs were spoken by a male voice and were presented at 65 dB-A in speech-shaped noise (SSN), with the initial SNR of +6 dB. Participants were instructed to select the consonant they heard from a presentation grid. VCV SRT-50 was obtained as for the BKB-SIN. Both tests were run in MATLAB R2013a (MathWorks). All the stimuli were presented via a Focusrite soundcard (Focusrite Audio Engineering Ltd, High Wycombe, UK), to a Fostex Personal Monitor 6301B loudspeaker (Fostex Company—a division of Foster Electric Co., Ltd., Tokyo, Japan), which was situated at 0° azimuth and 1.3 m from the participants’ HA microphone, in a double-wall soundproof booth. The methodology of SIN tests is described fully in [6]. The choice of using BKB-SIN and VCV in noise as SIN tests was motivated to evaluate two different stages of speech processing [29]. Since VCV have no contextual cues, it was a sublexical task demanding only consonant recognition. Instead, BKB-SIN was based on sentences with high-context, therefore, participants may predict words using top-down processing. Since the HA outcomes and benefits are measured with SIN performance, which, in turn, we aimed to predict with speech-ABRs, the aided speech-in-noise measures were used separately as dependent variables.

#### Self-report measure

Self-reported hearing status with HAs was measured using the Speech Spatial and Qualities of Hearing Questionnaire (SSQ; [1]). The SSQ contains three subscales: speech hearing, spatial hearing, and qualities of hearing. Because the current study aimed to predict self-reported speech understanding with Speech-ABRs as it was previously shown [30], only average participant ratings on SSQ-Speech subscale was used as a dependent variable.

#### Digit-span test

Each participant performed the Digit-Span Forward and Backward (DS-F and DS-B). The digit-span test is a subtest of the Wechsler Adult Intelligence Scale [31]. Short-term memory and working memory are assumed to be assessed via DS-F and DS-B, respectively. Füllgrabe et al. [32] reported a significant correlation between both DS-F and DS-B and speech recognition in noise. In order to control for short and working memory and based on previous results indicating a relationship to SIN performance, the DS-F and DS-B were used as possible predictors.

### Speech-ABRs

#### Equipment and recording parameters

Speech-ABRs were collected with Cambridge Electronic Design (CED, Cambridge, UK) Signal software (Version 5.11) using a CED power 1401 mkII data acquisition interface (CED Limited) and a Digitimer 360 isolated eight-channel patient amplifier (Digitimer Limited, Hertfordshire, UK). CED Signal software sampling configuration was set to gap-free sweep mode, sample rate of 20000 Hz, pulses with a resolution of 0.01 ms as the output type, and outputs were set at absolute levels and absolute times. Online second-order Butterworth filtering was set at 100 Hz (high-pass filter) and 3000 Hz (low-pass filter). Online artefact rejection was set to reject epochs that included any activity above 30 μV. Two-channel vertical electrode montage recording with Cz active, earlobe reference (A1 and A2), and high forehead ground (Fz) was used; electrode sites were based on the international 10–20 EEG system. Participants were laying in a comfortable recliner in a double-wall soundproof booth and were instructed to remain relaxed with their eyes closed. Loudspeaker positioning was at 45° azimuth, 1.1 m away from the participant’s aided ear. The different loudspeaker angle between the SIN tests and Speech-ABRs (0° vs. 45° azimuth) was due to the different position of the participants (seated upright vs. supine position), and that the loudspeaker could not be mounted from the ceiling. The different angles were unlikely to have affected the results.

#### Stimuli and recording procedure

The stimulus was a five-formant synthesized 40 ms [da] consisting of an onset burst within the first 10 ms and a vowel formant transition period with a rising F0 (103–125 Hz), rising first formant (220–720 Hz), falling second (1700–1240 Hz) and third formant (2580–2500 Hz), and constant fourth (3600 Hz) and fifth formants (4500 Hz). The 40 ms [da] was presented at 70 dB-A at a rate of 9.1 stimuli per second from the CED Signal software through the CED power 1401 mkII and routed through a Tucker-Davis Technologies (TDT, Alachua, FL, USA) PA5 programmable attenuator and a TDT HB7 headphone driver to a Fostex personal monitor 6301B loudspeaker (Fostex Company-a division of Foster Electric Co., Ltd., Tokyo, Japan). Stimulus polarity was reversed using Adobe Audition CC (2015.1 Release, build 8.1.0.162) in order to evoke speech-ABRs using two opposite stimulus polarities, each stimulus polarity was recorded separately. Speech-ABRs in noise were measured using a two-talker babble masker at 10 dB signal-to-noise ratio (SNR) background noise that was presented from Audacity (version 1.2.6) via an E-MU 0202 sound card (Creative Technology Limited, UK) and routed through the TDT HB7 headphone driver to the Fostex personal monitor 6301B loudspeaker; splitters were used in order for the stimuli and noise to be presented through the same loudspeaker. Speech-ABRs in quiet and in noise were recorded with (aided) and without (unaided) the HA. Two blocks of 2500 epochs (repetitions) were collected at each stimulus polarity (i.e. 5000 for polarity) for each of the four conditions (aided-quiet, aided-noise, unaided-quiet, and unaided-noise) for a total of 10.000 epochs per condition. Two blocks of each polarity were averaged separately and then baseline corrected via de-meaning to create two subaveraged alternating polarity responses.

#### Speech-ABR analyses

For each of the 4 conditions, peak latencies and amplitudes as well as the F0 encoding were extracted from the ABR waveform. Specifically, latencies for the positive speech-ABR peak V and negative peaks A, D, E, F, and O were measured. Amplitudes were measured for peak V to trough A (VA amplitude), and for negative peaks D, E, F, and O, the positive peak preceding each negative peak was used for peak to trough amplitude measurements. A speech-ABR peak was considered present if it was above the EEG noise floor with 95% confidence (as assessed with the bootstrap method; [33]) and if it was detected in the subaverages. Criteria and methods for peak identification are fully described in [6]. Once the F0 waveform of the 40 ms [da] was extracted as in [34], the envelope of the complex cross-correlation between speech-ABRs and the F0 waveform was estimated; the amplitude and latency of the envelope peak were taken as metrics and termed F0 encoding amplitude and latency, respectively (for further details see [6]).

### Decision trees

In the original work, BinKhamis et al. [6] included only aided speech-ABR F0 encoding amplitude and latency as possible predictors in the regression model. However, these two variables represent only a part of the information that is encoded in ABRs. Since regression trees are robust with missing data and can handle highly correlated predictors, it was possible to extend the analysis including also unaided speech-ABR F0 encoding and aided and unaided speech-ABR peaks. In this way, each of the three behavioural measures was used to fit two regression trees. The purpose was to explore all possible predictors.

Six regression trees were fitted to evaluate if the BKB-SIN, VCV and SSQ-Speech could be predicted using either speech-ABR peaks or speech-ABR F0 encoding. The non-ABR variables included in each tree were: participant age, DS-F, DS-B, and PTA; then three trees were fitted with amplitudes and latencies of speech-ABR peaks, and the other three trees were fitted with F0 encoding amplitude and latency. BinKhamis et al. [6] evaluated three multiple linear regression models using only speech-ABR aided-quiet and aided-noise conditions. Here, we used the data from all four speech-ABR conditions (aided-quiet, aided-noise, unaided-quiet, and unaided-noise).

All the regression trees were performed in R (version 3.6.3, [35]) using the package *rpart* [20], which implements the classical classification and regression tree algorithms developed by Breiman et al. [14]. The complexity parameter was set 0.0001 allowing growing a large tree [14, 15], which then was pruned obtaining the smallest tree that minimized the cross-validation error. The other parameters of the fitting were set to their default values, i.e., the number of cross-validations was 10 [14, 20], the minimum number of observations in any terminal node was taken as seven [20], and surrogate splits were used as recommended by Breiman et al. [14].

We had data from 92 participants. In order to obtain as stable as possible trees, it was decided to fit the trees with all observations, and to use the internal cross-validation procedure to evaluate the performance of the trees [20]. The cross-validation estimated the relative error and cross-validation error (xerror) of each tree. The latter is used for pruning because it is related to the predicted residual error sum of squares (PRESS) statistic [20, 36, 37]. The relative error is a measure of accuracy of the tree that, using the linear regression analogy, is equal to the 1-R^2^, which is the proportion of variance not explained by the model [14, 20]. The Root-Mean Square Error (RMSE) between the actual and predicted behavioural measures was calculated. Furthermore, the variable importance for each tree was also evaluated. The measure of importance is based on the sum over all nodes of the decrease of the error produced by splits of a given variable [14, 20]. For an easy interpretation, the importance of the variables was scaled to sum 100 [20].

## Results

**Fig 2:**
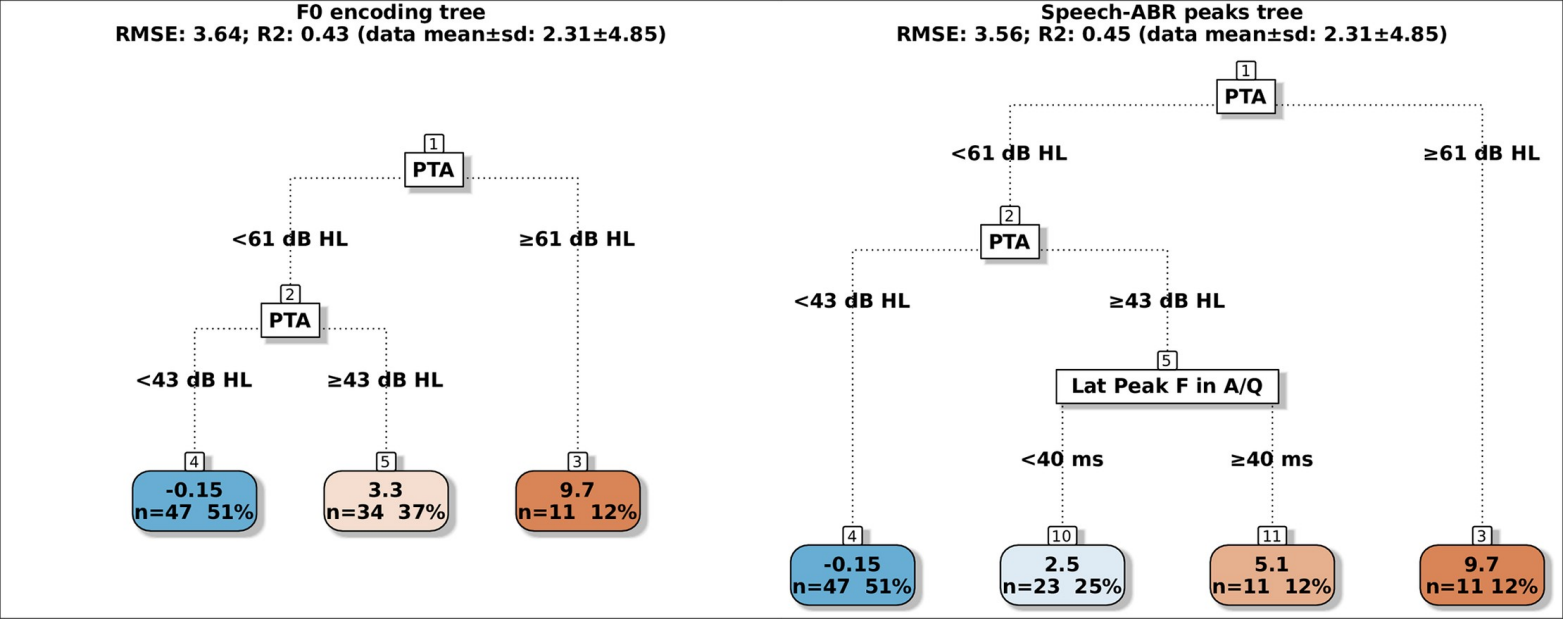
Regression trees for predicting the BKB-SIN SRT-50 using either F0 encoding measures (left tree) or speech-ABR peaks (right tree). Lat: latency; A/Q: aided speech-ABRs in quiet. The colour code of the terminal nodes, from blue to red, indicates worsening in participant performance.

**Fig 3:**
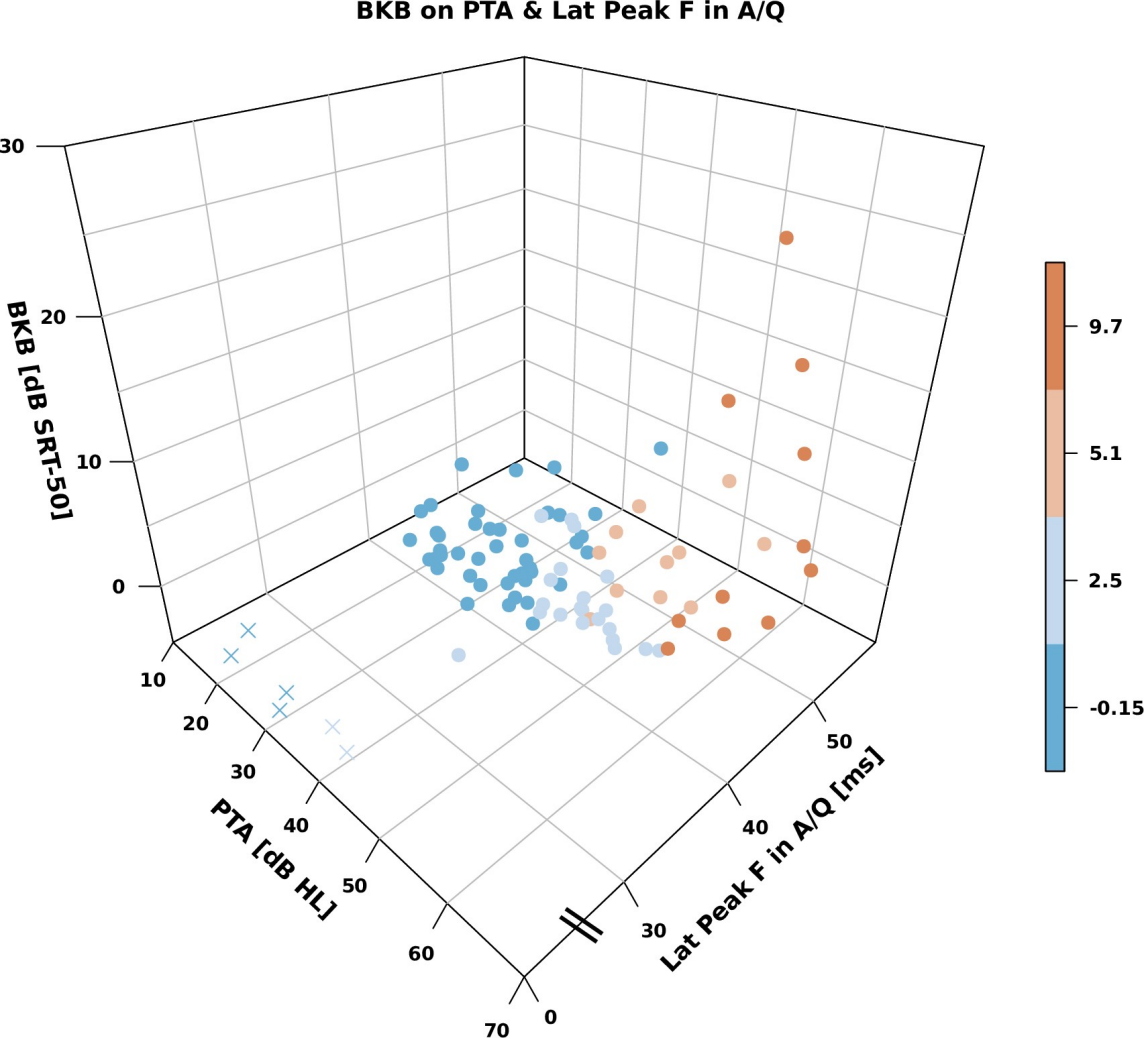
Regression trees for predicting the VCV SRT-50 using either F0 encoding measures (left tree) or speech-ABR peaks (right tree). Lat: latency; Amp: amplitude; A/Q: aided speech-ABRs in quiet. The colour code of the terminal nodes, from blue to red, indicates worsening in participant performance.

**Fig 4:**
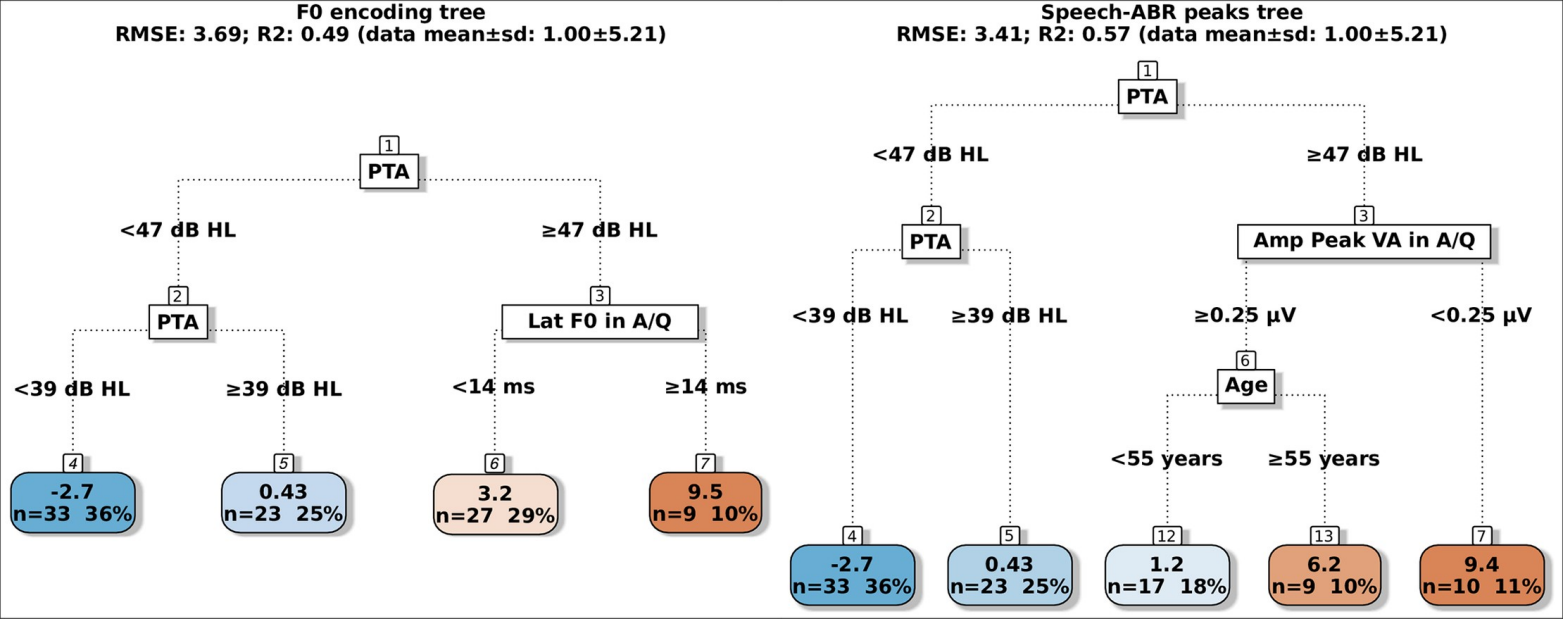
Regression trees for predicting the SSQ-Speech score using either F0 encoding measures (left tree) or speech-ABR peaks (right tree). The colour code of the terminal nodes, from blue to red, indicates worsening in participant performance.

**Fig 5:**
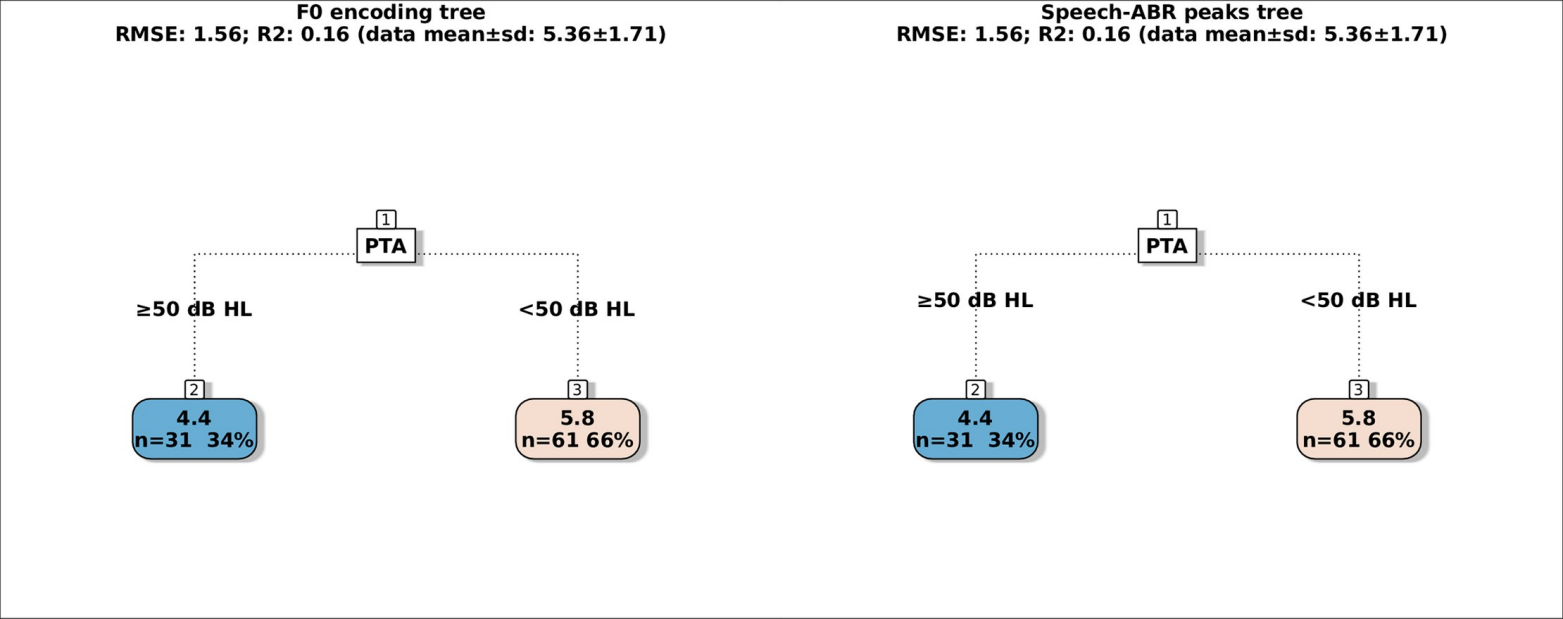
Relationship between BKB-SIN, PTA and aided latency of peak F. Missing peaks are indicated with crosses at 0 ms for graphical convenience. The colour code is the same as used in Fig 2.

The mean ± 1 SD of the dependent variables were 2.31±4.85 dB SRT-50 for the BKB-SIN, 1.00±5.21 dB SRT-50 for the VCV and 5.36±1.71 score for the SSQ-Speech. Scatter plots of the dependent variables as a function of PTA, the amplitudes and latencies of F0 encoding and speech-ABR peaks are in the supplement (Figs 2–8 in S1 File), these reveal that relationships between the behavioural measures and several speech-ABR peaks or F0 encoding cannot be explained by simple linear models. The regression trees for the BKB-SIN are shown in Fig 2, for the VCV in Fig 3, for the SSQ-Speech in Fig 4. Each figure shows trees using either F0 encoding (left panels) or speech-ABR peaks (right panels) among the predictor variables. Each terminal node gives the mean of the performance for that behavioural measure, and the number and percentage of participants within the node. The colour code of the terminal nodes, from blue to red, indicates worsening in participant performance. Table 1 in S1 File shows a summary of the trees’ parameters, and Table 2 in S1 File shows the three most important variables for each tree.

### BKB-SIN trees

In the F0 encoding tree (R^2^ = 0.43; RMSE = 3.64), there were three terminal nodes and two splits, both of which were based on PTA. The tree predicted that the mean BKB-SIN was -0.15 dB SRT-50 for the participants with PTA < 43 dB HL (47 participants, 51% of the total), 3.3 dB SRT-50 for those with PTA between 43 and 60 dB HL (34, 37%), and 9.7 SRT-50 for those with PTA ≥ 61 dB HL (11, 12%). The three most important variables were PTA, aided Fo encoding amplitude in quiet and then in noise (Fig 9 in S1 File). In the speech-ABR peaks tree (R2 = 0.45; RMSE = 3.56), the first two splits were based on PTA and were identical to the splits in the F0 encoding tree; however, the right branch of the second node (PTA between 43 and 60 dB HL) was further split by aided latency of peak F in quiet as the participants with peak F < 40 ms had mean of 2.5 dB SRT-50 (23, 25%), whereas the participants with peak F ≥ 40 ms had a mean of 5.1 dB SRT-50 (11, 12%). Fig 5 shows the relationship between BKB-SIN, PTA and aided latency of peak F. The three most important variables were PTA, aided amplitude of peak VA in noise, aided amplitude of peak E in quiet (Fig 10 in S1 File).

### VCV trees

In the F0 encoding tree (R^2^ = 0.49; RMSE = 3.69), there were four terminal nodes given by the split of the PTA and aided latency in quiet. The participants with PTA < 39 dB HL showed a mean performance of -2.7 dB SRT-50 (33 participants, 36% of the total), with PTA between 39 and 46 dB HL had mean performance of 0.43 dB SRT-50 (23, 25%), participants with PTA ≥ 47 dB HL and F0 encoding latency < 14 ms in quiet had mean performance of 3.2 dB SRT-50 (27, 29%), whereas the 9 participants with later latency ≥ 14 ms had mean performance of 9.5 dB SRT-50. The three most important variables were PTA, aided F0 encoding amplitude and latency in quiet (Fig 11 in S1 File). In the speech-ABR peaks tree (R2 = 0.57; RMSE = 3.41), the first and second nodes were PTA and were identical to the F0 encoding tree, the split of the third node was due to the aided amplitude of peak VA in quiet at 0.25 μV. The left branch (i.e. ≥ 0.25 μV) was further split by age where participants younger than 55 years had mean performance of 1.2 dB SRT-50 (17, 18%) while the older ones had mean performance of 6.2 dB SRT-50 (9, 10%). The 10 participants with amplitude < 0.25 μV had mean performance of 9.4 dB SRT-50. After PTA, the next most important variables were the aided amplitude of peaks VA and E in quiet (Fig 12 in S1 File).

### SSQ-Speech trees

Both the F0 encoding and speech-ABR peaks tree (R2 = 0.16; RMSE = 1.56) had only one node that split the participants by PTA at 50 dB HL where worse hearing thresholds predicted an average score of 4.4 (31 participants, 34% of the total) while the better hearing thresholds predicted an average score of 5.8 (61, 66%). After PTA, the next most important variables were F0 encoding aided amplitude in quiet and unaided latency in quiet in the F0 encoding tree (Fig 13 in S1 File); and the aided amplitude of peak E in quiet and aided amplitude of peak F in noise in the speech-ABR peaks trees (Fig 14 in S1 File).

## Discussion

### Accuracy of the regression trees

In order to evaluate whether the regression trees were accurate or, in other words, worthwhile [38], two metrics are generally used: Root Mean Square Error (RMSE) and R^2^ [15]. The RMSE measures the lack of fit of the model relative to the data [15]. Since the MSE can be broken down into variance and squared bias of the data [15, 21], in the case of unbiased estimator of the mean, the MSE of the model and variance of the data are the same [15], and so are their square roots, namely RMSE and standard deviation (SD). However, a predictor with low bias may have high variance (that is the bias-variance trade-off, [15]). Nevertheless, the RMSE of the model and SD of the data can be compared and, being the same unit, are easily interpretable. Instead, R^2^ is the proportion of variance explained by the model; it is a-dimension with a range between 0 and 1.

The SSQ-speech trees were root node trees, i.e., with only the root, which lead to a poor accuracy as the RMSE was slightly better than SD (1.56 vs. 1.71 score) and the variance accounted by the trees was small (R^2^ = 0.16).

Considering the trees for the BKB-SIN and VCV, their RMSE’s were below 4 dB SRT-50 and smaller relative to the standard deviation of the behavioural measures. Looking at the proportion of variance explained by the trees, the values of R^2^ laid between 0.43 (BKB-SIN with F0 encoding) and 0.57 (VCV with the speech-ABR peaks). Overall, the regression trees had good accuracy. Other statistical learning algorithms, such as Random Forest [39] or Boosting [40, 41], may give better predictive accuracy than the regression trees [15]. However, the accuracy was not the critical objective of the current study, but rather we were interested to understand the structural relationships between behavioural and speech-ABR measures; the aim was to assess if speech-ABRs are a clinically applicable objective outcome measure in HA users.

### Variable importance

The most important variable was PTA in each of the six trees. This was in line with the results obtained by BinKhamis et al. [6], who studied a subset of our predictors. The first splits from the root were always due to PTA. This suggests that PTA is more informative than any of the speech-ABR measures.

Putting to one side the SSQ-Speech trees as they had only one node, we extrapolated the three most important variables for each tree, for a total of 12 variables. Of the eight variables with speech-ABR measures, six were in the quiet condition and two in background noise, and all were aided. So, the most informative condition was aided quiet. The SIN tests were performed aided and in noise. The trees may rely mainly on quiet condition because the speech-ABR in quiet is more detectable than in noise [6, 7] as the former condition allowed to have both more inter-subject variability (i.e. more information available for modelling) and less missing data points relative to the speech-ABR in noise.

Some of the variables ranked as important but were not used in the trees. In the F0 encoding VCV tree, the aided F0 encoding amplitude, despite not being a node, was more important than the aided F0 encoding latency, which was a tree node. This was because, in the root node, the optimal split was given by PTA while the best suboptimal split was due to the F0 encoding amplitude. So, being suboptimal has contributed to its importance [14].

### Prediction of behavioural measures with speech-ABRs

Two studies were able to correlate SIN tests performance with F0 amplitude estimated in the Fourier domain from the steady-state region of the 170-ms [da]. It was reported that F0 amplitude in quiet for older adults (mean 63.1 years old) and F0 amplitude in noise for young adults (mean 24 years old) were larger for the participants with better SIN tests performance relative to those with worse performance [42, 43]. The older adults had normal or near-normal hearing up to 4 kHz and some degree of hearing loss at higher frequency [42], whereas the young adults had normal hearing [43]. Others did not find the association between speech-ABRs and SIN test performance in adults with normal or near-normal absolute thresholds up to 4 kHz. For instance, the correlation between F0 through the fifth harmonic recorded in older adults (mean 70 years old, and audiometric thresholds ≤ 20 dB HL up to 4 kHz) and SIN tests performance was not significant [44]. Interestingly, Coffey et al. [45] reported a positive correlation between F0 and SIN tests using magnetoencephalography (MEG) but not using EEG in normal-hearing adults. In one of the few studied with HA users, Easwar et al. [12] observed a positive correlation between EFR amplitudes and both speech discrimination scores and sound quality ratings. Seol et al. [46] investigated the effects of HA noise reduction algorithms for speech understanding and speech-ABRs both in quiet and in noise. They reported a positive correlation between F0 amplitudes and SIN tests performance irrespective of the background (quiet or noise), however it was not clear how the speech-ABRs were recorded (unaided or aided, cf. their Figs 5 and 6) or how the presence/absence of a speech-ABR was established as several F0 amplitudes were close to 0 μV. In the current study, the F0 encoding latency in quiet was a node in the VCV tree, splitting the performance of the participants with PTA ≥ 47 dB HL. The delayed F0 measures were associated with the nine poorest performers. Because our CV stimulus was shorter relative to those in the above-mentioned studies, our frequency resolution was lower and this may have an effect on the estimation of F0. Indeed, the EFR amplitude in [12] was the sum of the response amplitudes across the eight formant carrier of the stimulus, whereas our F0 was based on one carrier. This may explain the lack of F0 measures as a strong predictor in our case. Furthermore, stimuli with a sustained component (such as [susa∫i] or 170-ms [da]) can provide information about temporal fine structure, which might be important for speech perception in noise [47, 48]. However, a longer stimulus will result in a longer recording session [7], which has to be kept to a minimum for a clinical setting, and in the degradation of the temporal characteristics of speech-ABRs [5]. Overall, taken together, these mixed results do not clarify the role of F0 measures in the SIN prediction, and further studies are required, in particular involving HA users.

Beside the features extracted in the frequency domain, such as the F0 measures, the amplitude and latency of speech-ABR peaks are the key temporal features to evaluate. In this framework, Parbery-Clark et al. [49] examined speech-ABRs evoked by 170-ms [da] within multi-talker babble (the SNR was 10 dB as in this study) in normal-hearing adults and their performance on a SIN test. Using the speech-ABR peaks elicited by the stimulus onset (at 9–11 ms) and the formant transition period (at 43–45 ms) as features, they observed positive correlations between the latency of both peaks and SIN test performance. In the current study, the onset peak was labelled as peak VA. In the BKB-SIN tree, the amplitude of peak VA in noise was among the important variables. In the VCV tree, the amplitude of peak VA in quiet was a predictor for the subgroup of participants having the worst PTA and performance. Furthermore, their formant transition peak can be associated with our peaks D, E, and F [8, 9]. The amplitude of peak E in quiet was among the important variables for both the BKB-SIN and VCV trees. In the BKB-SIN tree, latency of peak F in quiet was a predictor only for the participants with PTA between 43 and 61 dB HL.

For a subgroup of participants, both the onset (i.e. peak VA) and the FFR (i.e. peaks E and F, and the F0 encoding measures) of speech-ABRs appeared to be relevant for SIN performance. The speech onsets and, in turn, the speech-ABRs onset, have been theorised to be linked to phoneme identification and language literacy [50]. Therefore, abnormal amplitude or timing may indicate less robust phoneme identification resulting in reduced speech understanding in noise. Abrams & Kraus [50] proposed that FFR measures represent frequency transitions, which are important for suprasegmental cues (e.g. speaker’s gender and emotion) and for consonant identification. Consequently, impairment in the FFR may be linked to a deficit in utilising these cues and, hence, to a difficulty to understand speech in noise.

The effects of age on speech-ABRs and SIN performance were examined by Heidari et al. [51] and Mamo et al. [44]. In both studies, the comparison between younger and older adults showed that the latter had worse SIN performance and lower speech-ABR amplitude than the younger groups. Age was among the predictors for the VCV in the speech-ABR peaks tree, but only when aided amplitude of peak VA in quiet was ≥ 0.25 μV. The VCV performance worsened as a function of age but without a strong group division as in the previous studies [44, 51]. Clinard & Tremblay [52] observed that the amplitudes of the onset responses (waves V and A) degraded significantly as age increased.

Regarding SSQ-Speech, Anderson et al. [30] reported that latency of the peak O (i.e. offset) and the ’response morphology’ (i.e., cross-correlation between stimulus and response waveforms) of speech-ABR were significant predictors for the SSQ-Speech score. As in our study, the speech-ABRs were evoked by a 40 ms [da]. Differently to the current study, Anderson et al. [30] obtained speech-ABR monaurally through insert earphones (we used loudspeakers) in adults with both normal-hearing and hearing loss (we had only adults with SNHL), having average PTA (0.5–4 kHz) from 2.5 to 44.5 dB HL (in our case from 12 to 68 dB HL). In their study, the PTA was not a significant predictor, instead, in the SSQ-Speech trees, the only node split the participants at a PTA of 50 dB HL, that is near their worst PTA. So, the two results may be reconciled assuming that as PTA has larger variance or range, it may give a stronger contribution to explain the SSQ-Speech score.

In the current study, the PTA range was wider relative to the above-mentioned studies. The wide range of PTA means that the PTA is the most relevant feature. However, the wide range of PTA is an asset here as it allowed assessing the speech-ABR as a possible objective measure for HA users in a clinically plausible scenario, given that a wide variability in PTA is common in a clinical setting.

### Non-linearity and sub-groups

One of the reasons for using regression trees was to disclose plausible non-linear boundaries between the predictors. Instead, the models predicted monotonic relations for BKB-SIN and VCV trees. Considering for instance the tree fitted to VCV with speech-ABR peaks: worse hearing threshold led to worse performances, younger participants outperformed the older ones, and smaller speech-ABR amplitudes were associated with worse performance. However, the tree allowed pinpointing the sub-groups of participants for which the prediction was based on speech-ABR features. In the case of the BKB-SIN tree, the aided latency of peak F in quiet can be used for the participants with PTA between 43 and 61 dB HL, this feasibility within a range of only 18 dB HL would not be useful as a clinical tool. In the case of the VCV trees, F0 encoding and speech-ABR measures split the participants with PTA ≥ 47 dB HL in two sub-groups, pointing-out the nine/ten participants with the poorest performance, as such a method aimed at a niche group of participants may not be clinically relevant but is promising for future research. It will also be worthy to explore if speech-ABRs evoked by other stimuli (e.g., longer than those used here) would model and predict behavioural measures in HA users with a wide range of PTA.

## Conclusion

We anticipated strong relationships between speech-ABR and behavioural measures and that some of them would be non-linear. To test these predictions, an existing dataset was re-analysed using regression trees. Importantly, the dataset is probably the largest available with both speech-ABR and behavioural measures in HA users. Although a clinical or uniform standard for speech-ABRs recording and processing is not specified, in order to make the most of data, we extracted several features of speech-ABRs that have been reported in the literature. Our results demonstrated that relations between speech-ABR and behavioural measures were present only for a small subset of the participants, and they were monotonic. Overall, the most relevant feature to predict behavioural measures was the PTA.

## Supporting information

S1 File Supporting information. (PDF)
