# Correction: On prediction of aided behavioural measures using speech auditory brainstem responses and decision trees

**DOI:** 10.1371/journal.pone.0307864

**Published:** 2024-07-23

**Authors:** The PLOS ONE Staff

In the Abstract, there is an error in the ninth sentence of the paragraph. The correct sentence is: In the VCV trees, performance was predicted by the aided F0 encoding latency and the aided amplitude of peak VA in quiet for participants with PTAs ≥ 47 dB HL.

In the Results, there is an error in the third sentence of the paragraph. The correct sentence is: The regression trees for the BKB-SIN are shown in Fig 2, for the VCV in Fig 4, for the SSQ-Speech in Fig 5.

In the BKB-SIN trees subsection of the Results, there is an error in the fifth sentence of the paragraph. The correct sentence is: Fig 3 shows the relationship between BKB-SIN, PTA and aided latency of peak F.

The captions for Figs 3–5 are incorrectly switched. The correct captions have been provided here:

**Fig 3:**
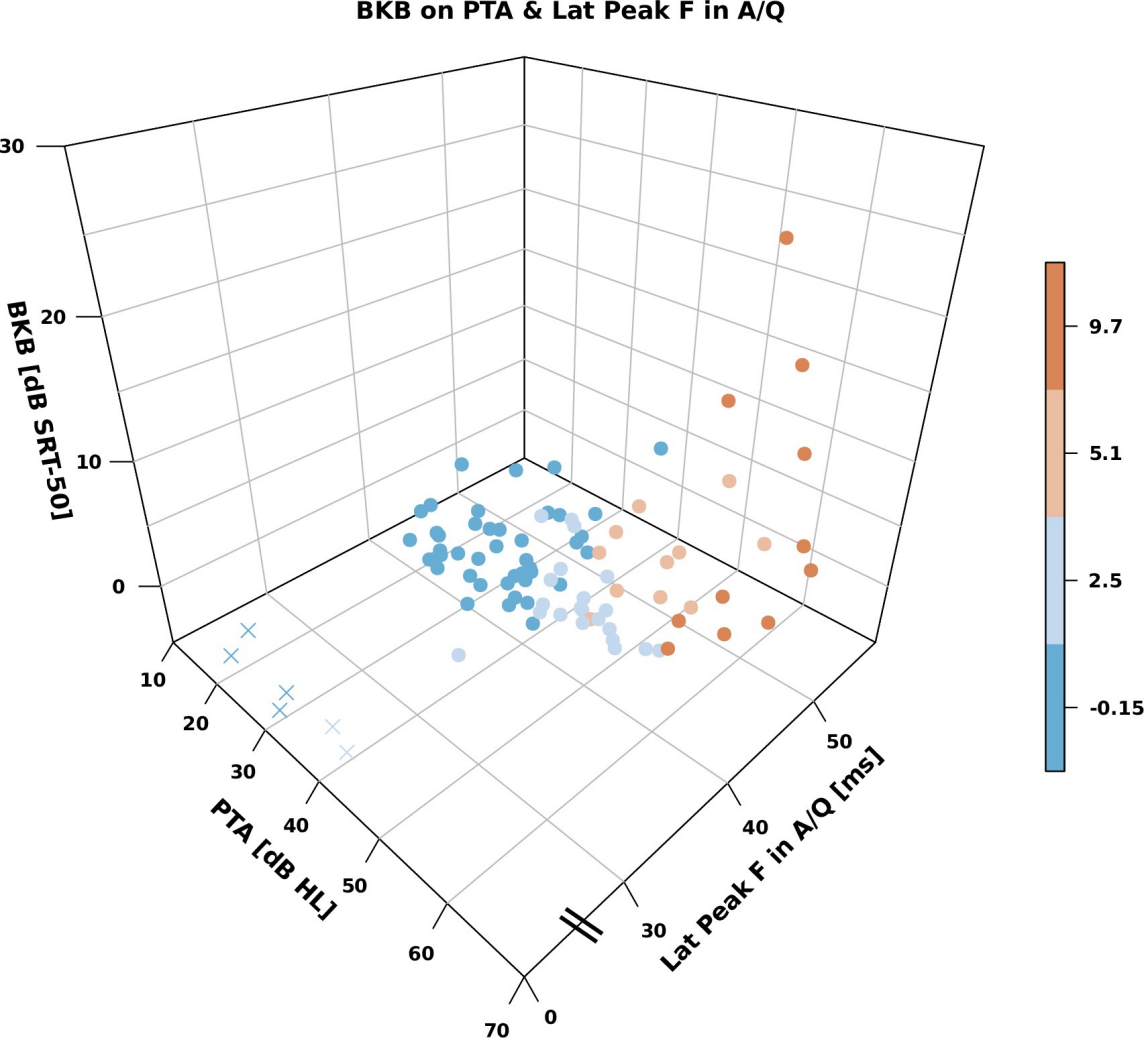
Relationship between BKB-SIN, PTA and aided latency of peak F. Missing peaks are indicated with crosses at 0 ms for graphical convenience. The colour code is the same as used in Fig 2.

**Fig 4:**
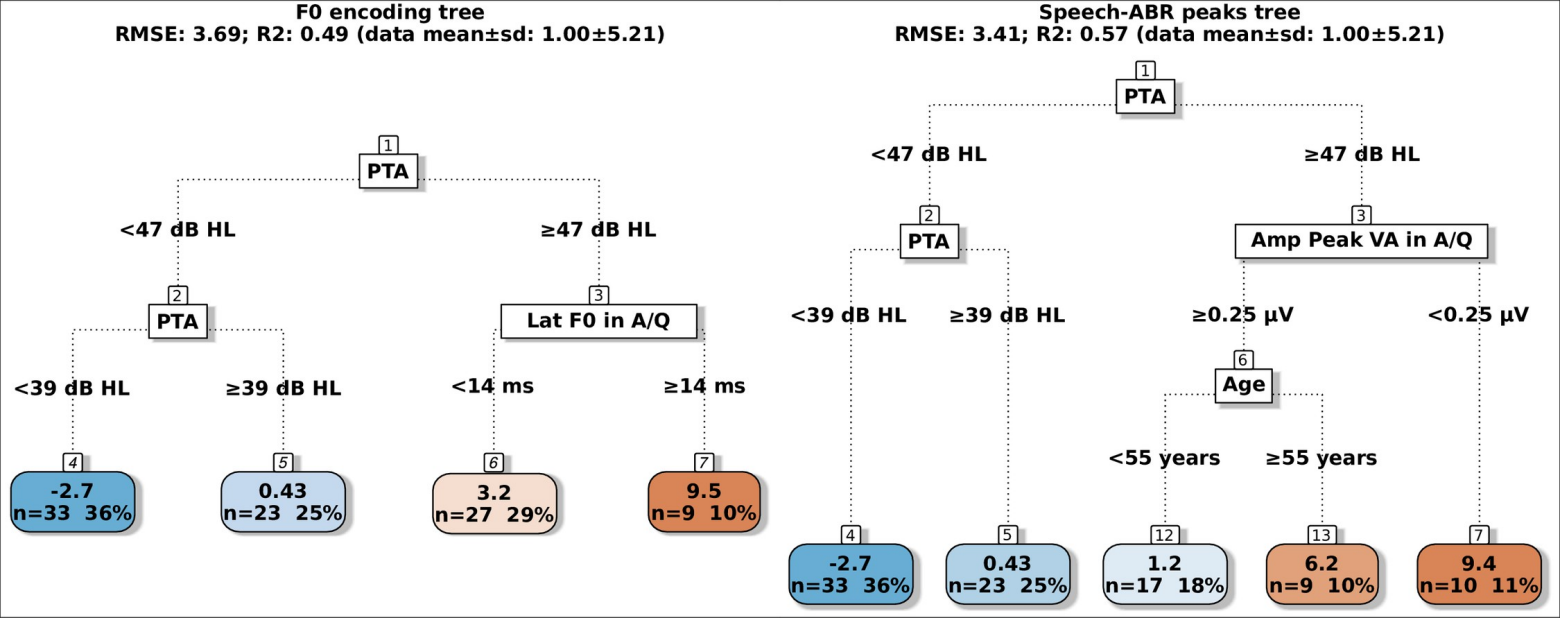
Regression trees for predicting the VCV SRT-50 using either F0 encoding measures (left tree) or speech-ABR peaks (right tree). Lat: latency; Amp: amplitude; A/Q: aided speech-ABRs in quiet. The colour code of the terminal nodes, from blue to red, indicates worsening in participant performance.

**Fig 5:**
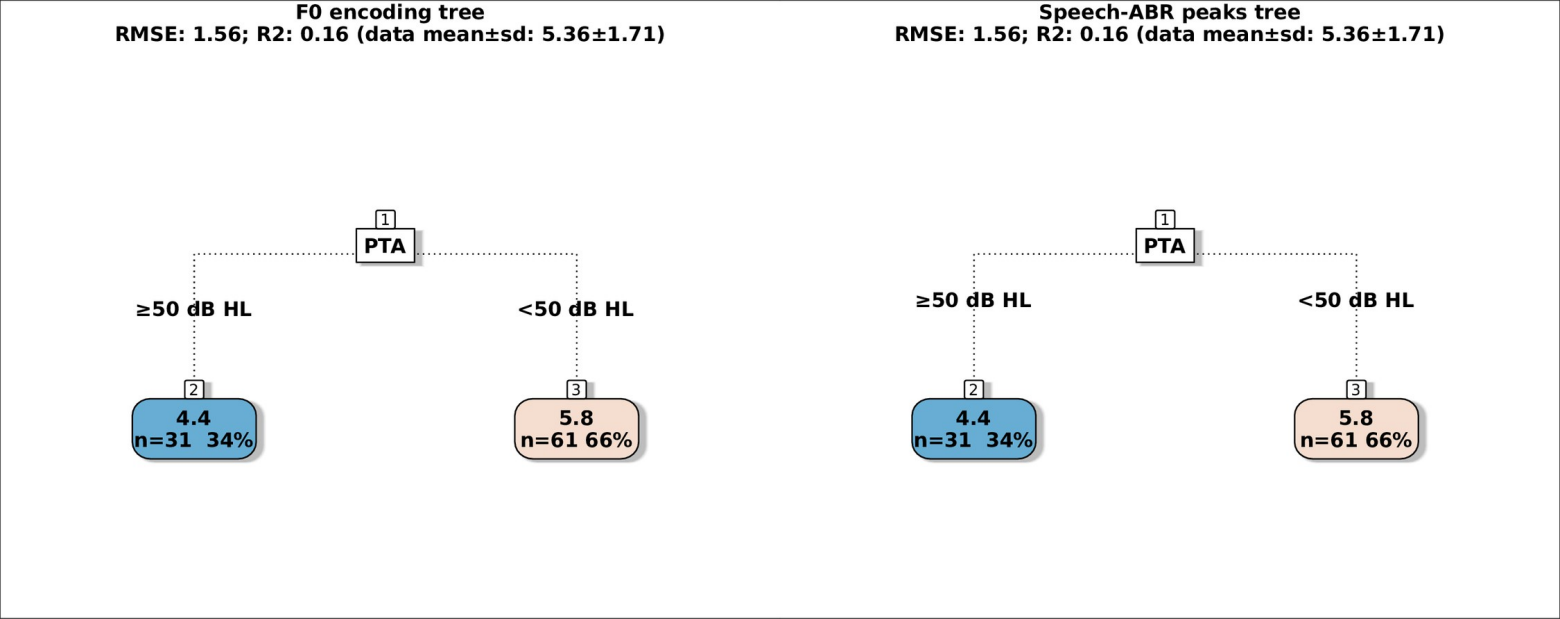
Regression trees for predicting the SSQ-Speech score using either F0 encoding measures (left tree) or speech-ABR peaks (right tree). The colour code of the terminal nodes, from blue to red, indicates worsening in participant performance.

The publisher apologizes for the errors.
