# Supplementary material for: On prediction of aided behavioural measures using speech auditory brainstem responses and decision trees
Source: PLoS One. 2021 Nov 16;16(11):e0260090. doi: 10.1371/journal.pone.0260090 (PMC8594837; doi:10.1371/journal.pone.0260090)
Supplement: S1 File — (PDF) [file pone.0260090.s001.pdf]

On prediction of aided behavioural measures using Speech Auditory Brainstem Responses and Decision Trees

Supporting Information

List of Figures

1 Distributions of participants' age. 2
2 BKB-SIN SRT-50, VCV SRT-50 & SSQ-Speech score on PTA 3
3 BKB-SIN SRT-50 on amplitudes of speech-ABR 4
4 BKB-SIN SRT-50 on latencies of speech-ABR 5
5 VCV SRT-50 on amplitudes of speech-ABR 6
6 VCV SRT-50 on latencies of speech-ABR 7
7 SSQ-Speech score on amplitudes of speech-ABR 8
8 SSQ-Speech score on latencies of speech-ABR 9
9 BKB-SIN SRT-50 on PTA & important variables for the F0 encoding tree 10
10 BKB-SIN SRT-50 on PTA & important variables for the speech-ABR peaks tree . . . 10
11 VCV SRT50 on PTA & important variables for the F0 encoding tree 11
12 VCV SRT50 on PTA & important variables for the speech-ABR peaks tree 11
13 SSQ-Speech score on PTA & important variables for the F0 encoding tree 12
14 SSQ-Speech score on PTA & important variables for the speech-ABR peaks tree 12

List of Tables

1 Regression trees summary table 13
2 Variable importance table 14

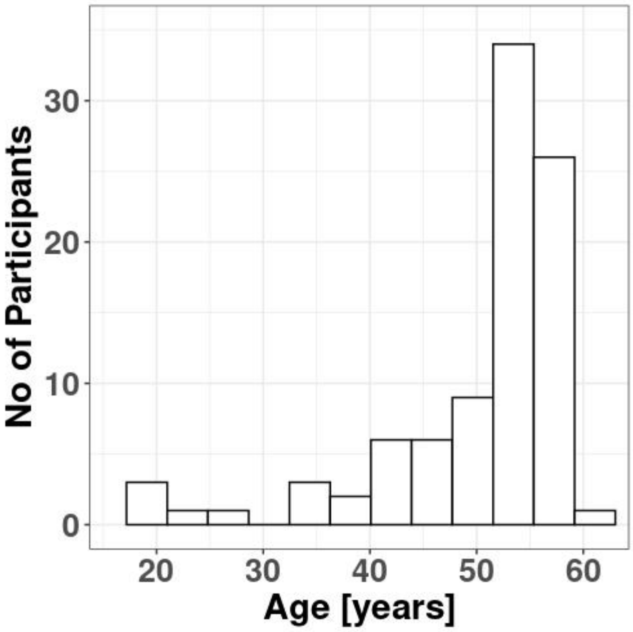

Figure 1: Distributions of participants' age.

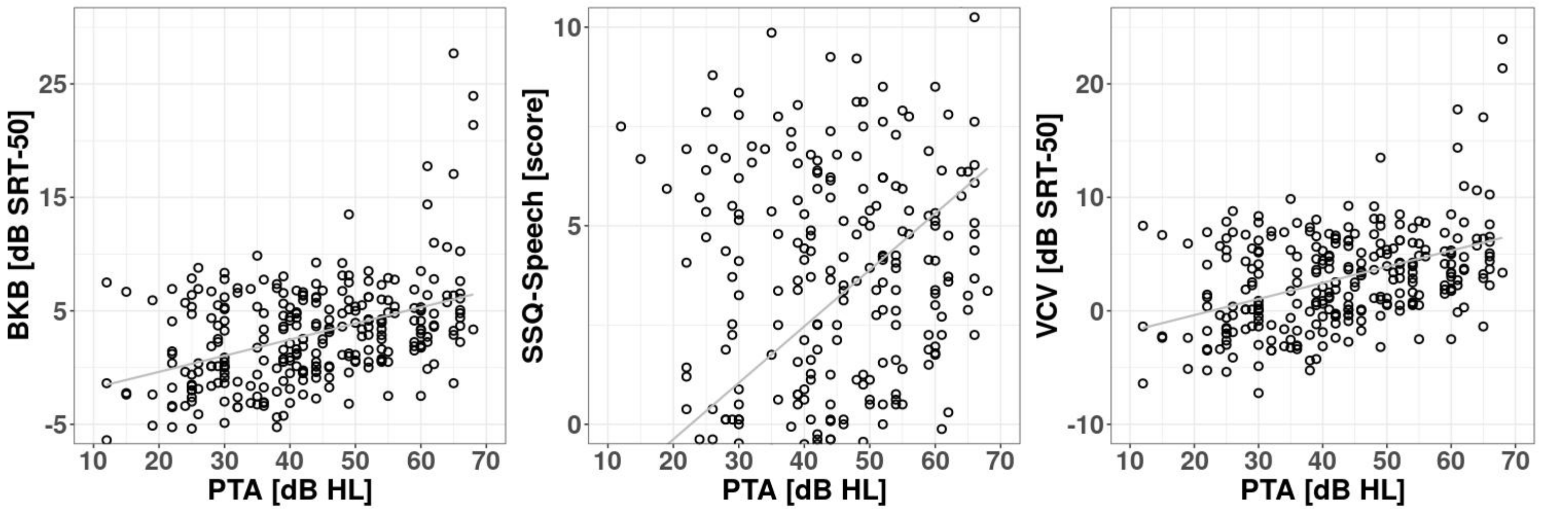

Figure 2: BKB-SIN SRT-50 (left panel), VCV SRT-50 (central panel), and SSQ-Speech score (rigth panel) as a function of PTA.

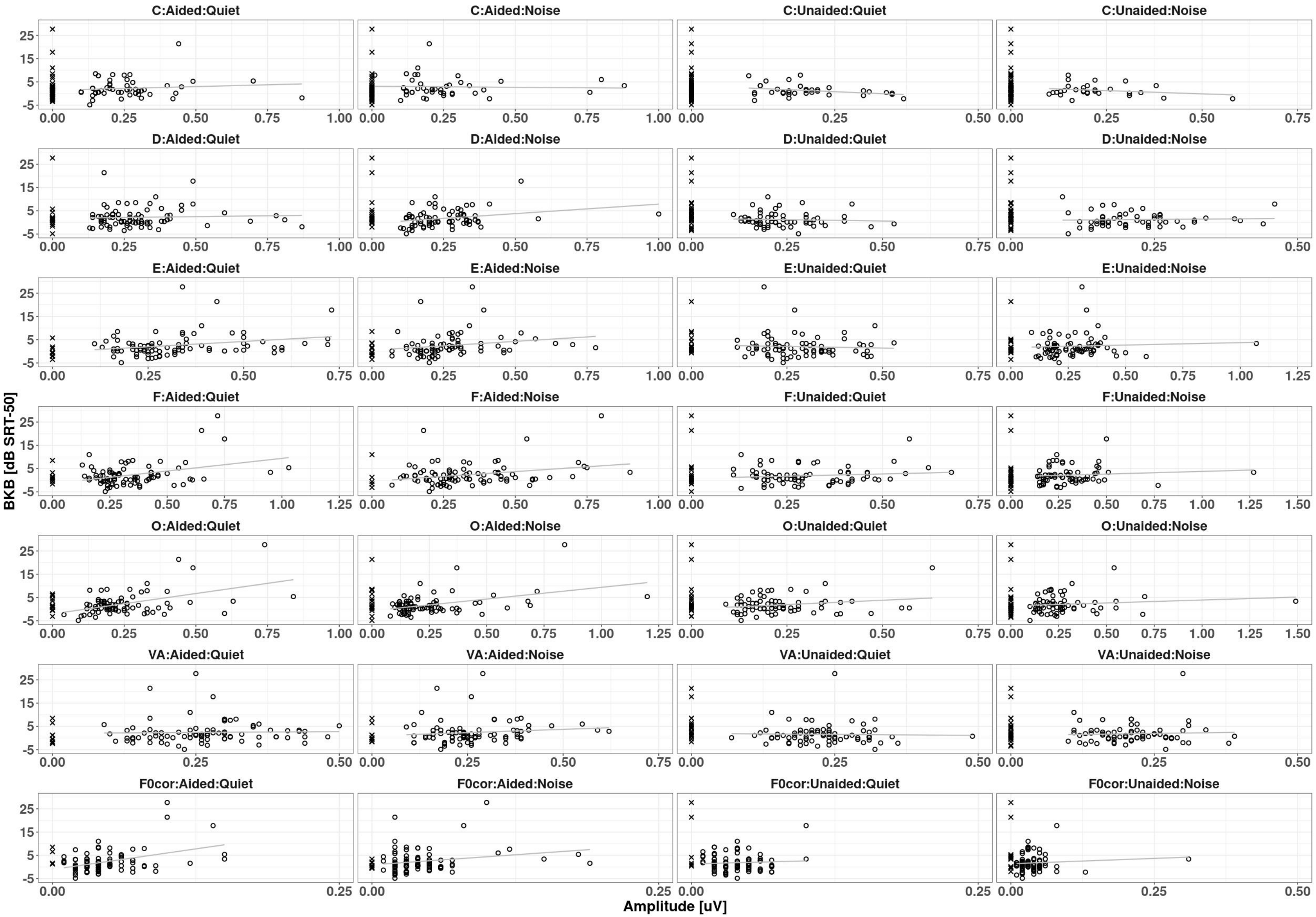

Figure 3: BKB-SIN SRT-50 as a function of the amplitudes of the speech-ABR peaks (i.e. *C*, *D*, *E*, *F*, *O*, *VA*) and F0 encoding (i.e. *F0cor*) in the 2x2 conditions (*Aided* vs. *Unaided* and *Quiet* vs. background *Noise*). Missing peaks and F0 encoding are indicated with crosses at 0 uV for graphical convenience (these data were used in the regression trees). Note: the x-axis scale is not equal across sub-plots.

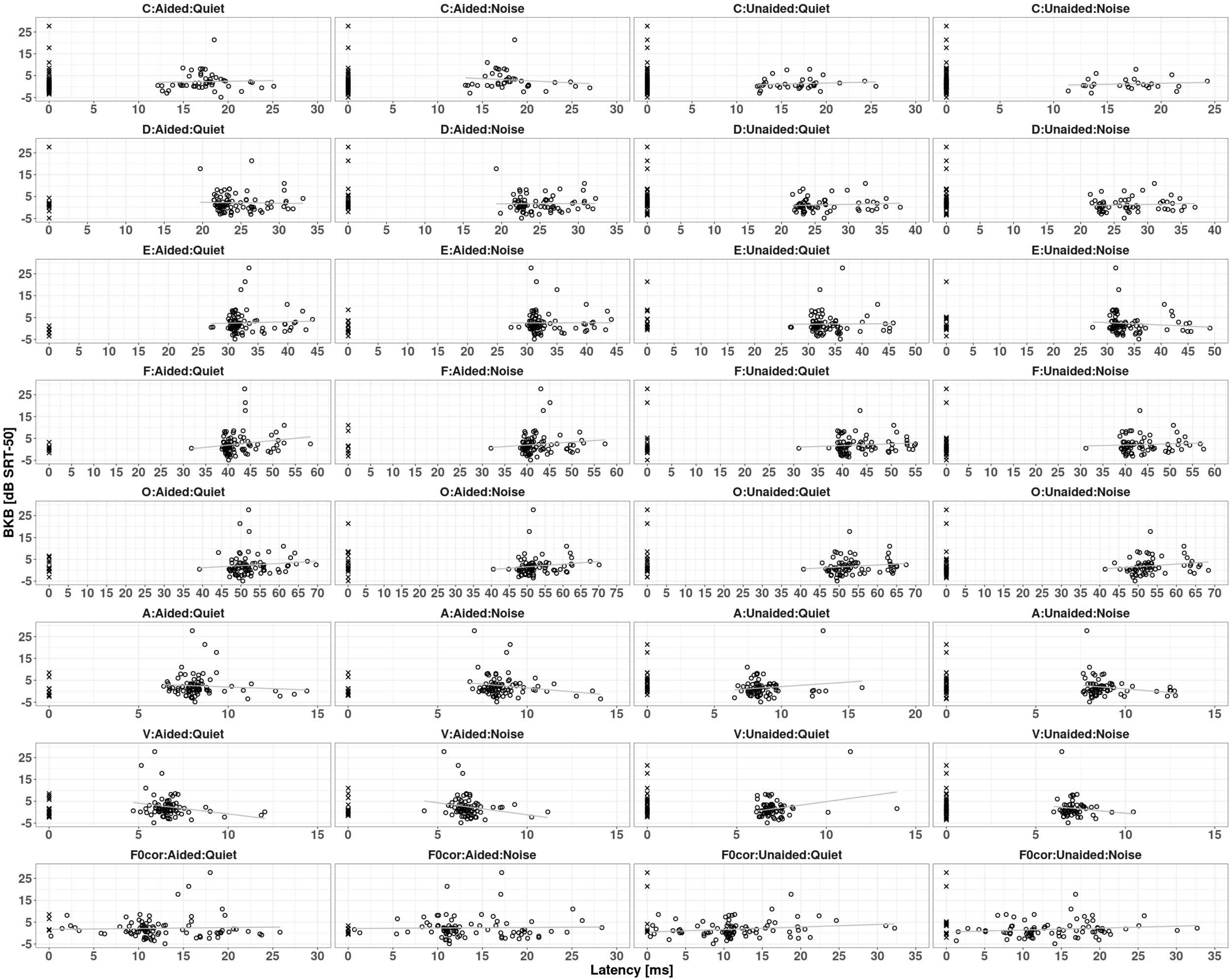

Figure 4: BKB-SIN SRT-50 as a function of the latencies of the speech-ABR peaks (i.e. *C*, *D*, *E*, *F*, *O*, *A*, *V*) and F0 encoding (i.e. *F0cor*) in the 2x2 conditions (*Aided* vs. *Unaided* and *Quiet* vs. background *Noise*). Missing peaks and F0 encoding are indicated with crosses at 0 ms for graphical convenience (these data were used in the regression trees). Note: the x-axis scale is not equal across sub-plots.

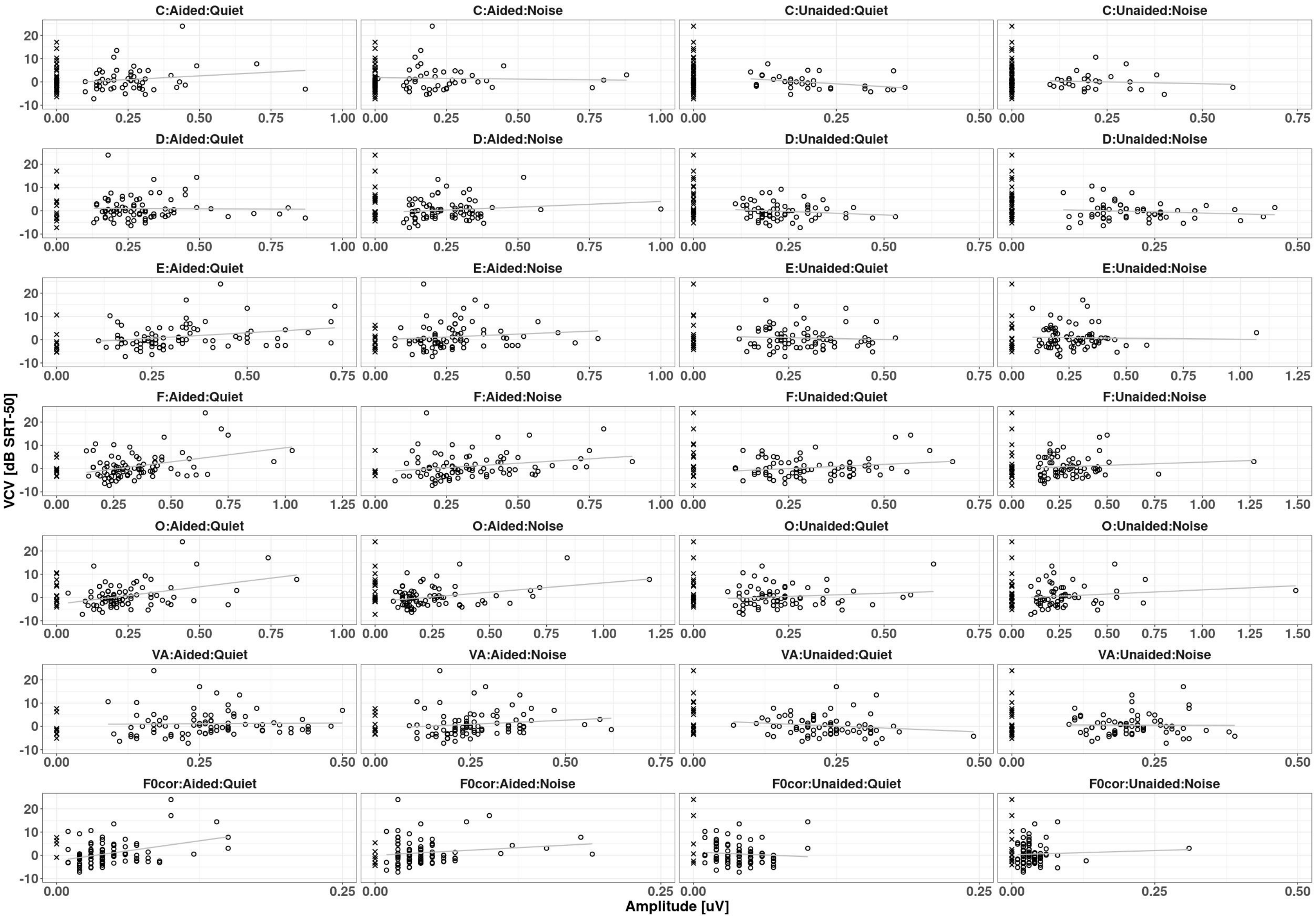

Figure 5: VCV SRT-50 as a function of the amplitudes of the speech-ABR peaks (i.e. *C*, *D*, *E*, *F*, *O*, *VA*) and F0 encoding (i.e. *F0cor*) in the 2x2 conditions (*Aided* vs. *Unaided* and *Quiet* vs. background *Noise*). Missing peaks and F0 encoding are indicated with crosses at 0 uV for graphical convenience (these data were used in the regression trees). Note: the x-axis scale is not equal across sub-plots.

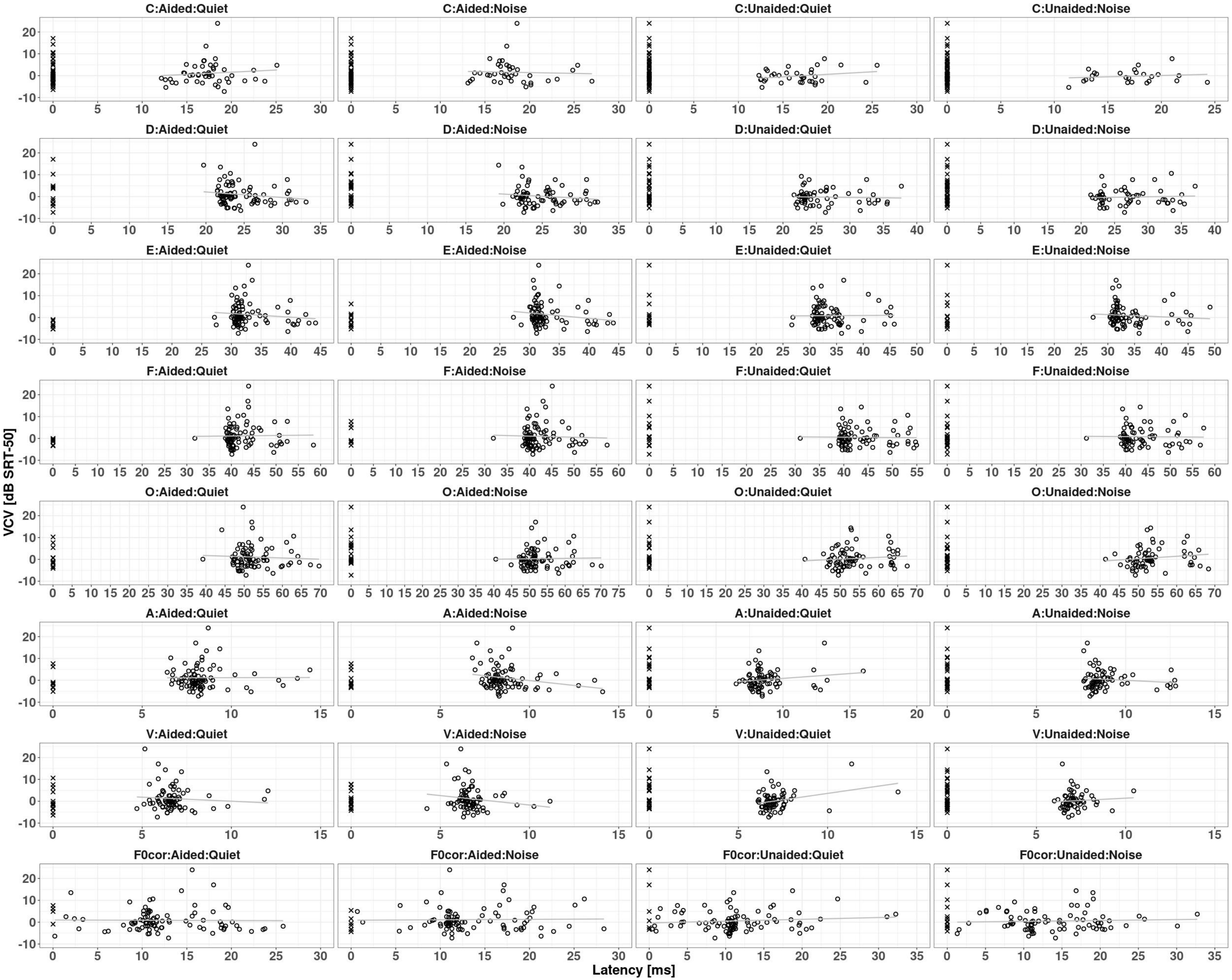

Figure 6: VCV SRT-50 as a function of the latencies of the speech-ABR peaks (i.e. *C*, *D*, *E*, *F*, *O*, *A*, *V*) and F0 encoding (i.e. *F0cor*) in the 2x2 conditions (*Aided* vs. *Unaided* and *Quiet* vs. background *Noise*). Missing peaks and F0 encoding are indicated with crosses at 0 ms for graphical convenience (these data were used in the regression trees). Note: the x-axis scale is not equal across sub-plots.

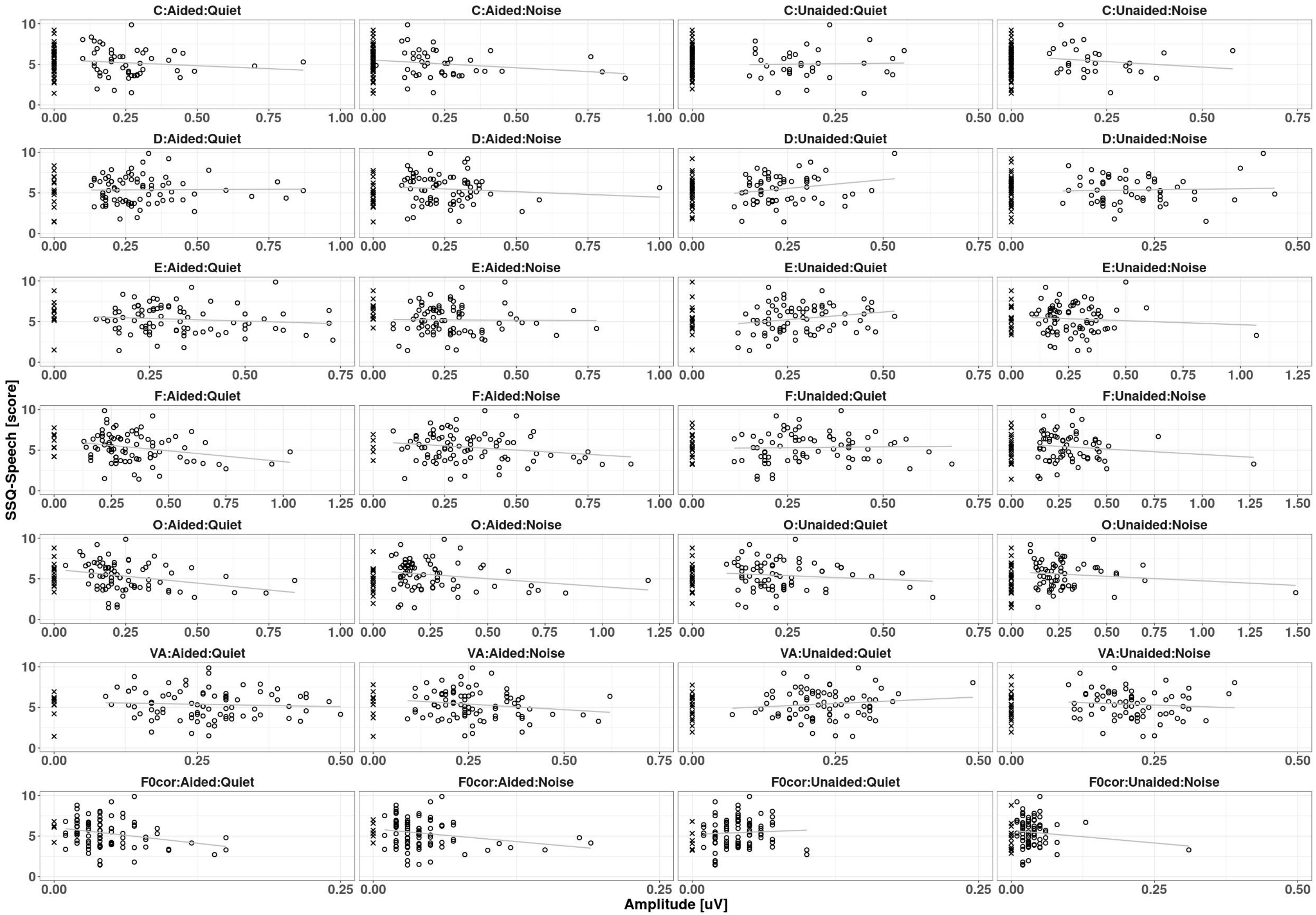

Figure 7: SSQ-Speech score as a function of the amplitudes of the speech-ABR peaks (i.e. *C*, *D*, *E*, *F*, *O*, *VA*) and F0 encoding (i.e. *F0cor*) in the 2x2 conditions (*Aided* vs. *Unaided* and *Quiet* vs. background *Noise*). Missing peaks and F0 encoding are indicated with crosses at 0 uV for graphical convenience (these data were used in the regression trees). Note: the x-axis scale is not equal across sub-plots.

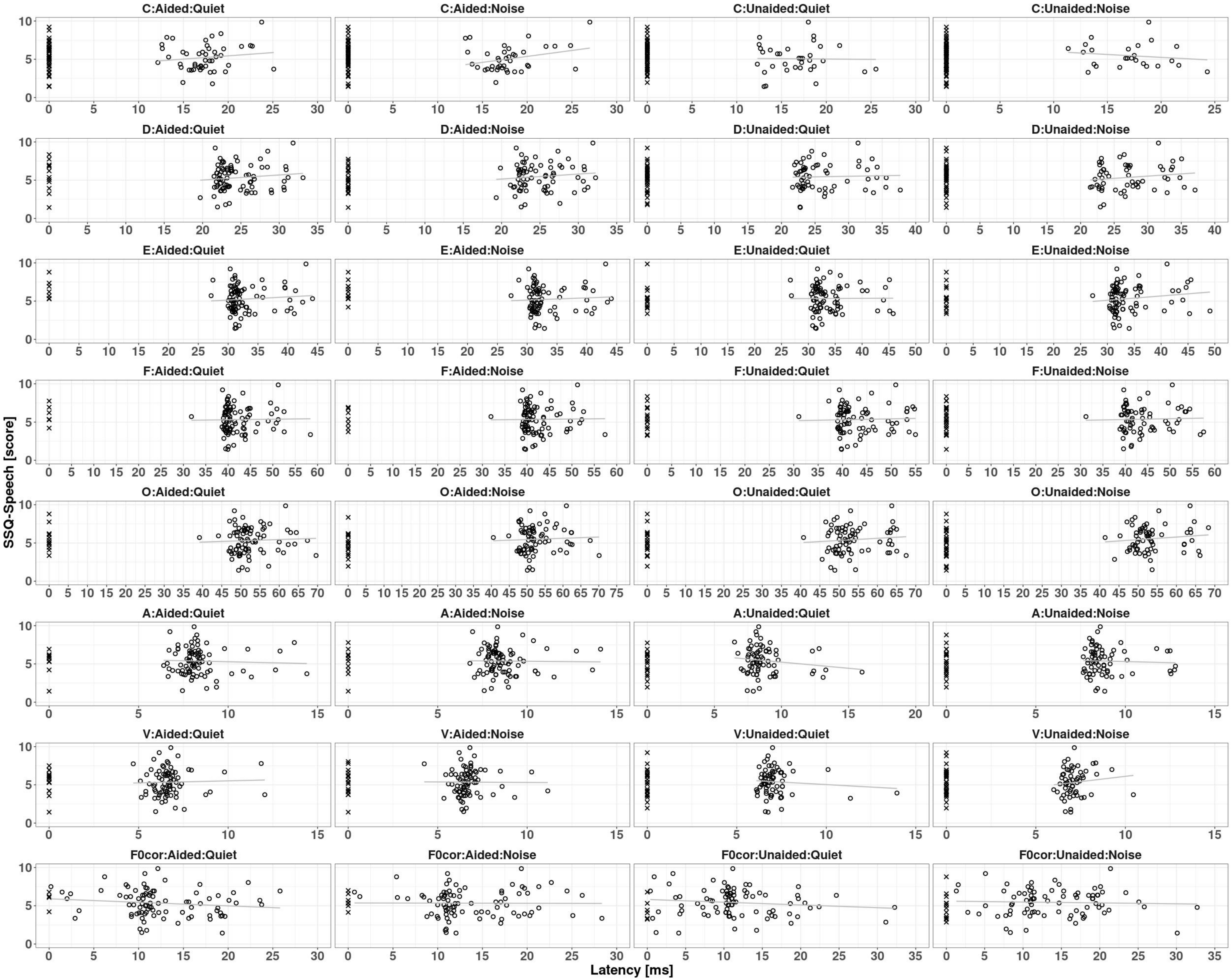

Figure 8: SSQ-Speech score as a function of the latencies of the speech-ABR peaks (i.e. *C*, *D*, *E*, *F*, *O*, *A*, *V*) and F0 encoding (i.e. *F0cor*) in the 2x2 conditions (*Aided* vs. *Unaided* and *Quiet* vs. background *Noise*). Missing peaks and F0 encoding are indicated with crosses at 0 ms for graphical convenience (these data were used in the regression trees). Note: the x-axis scale is not equal across sub-plots.

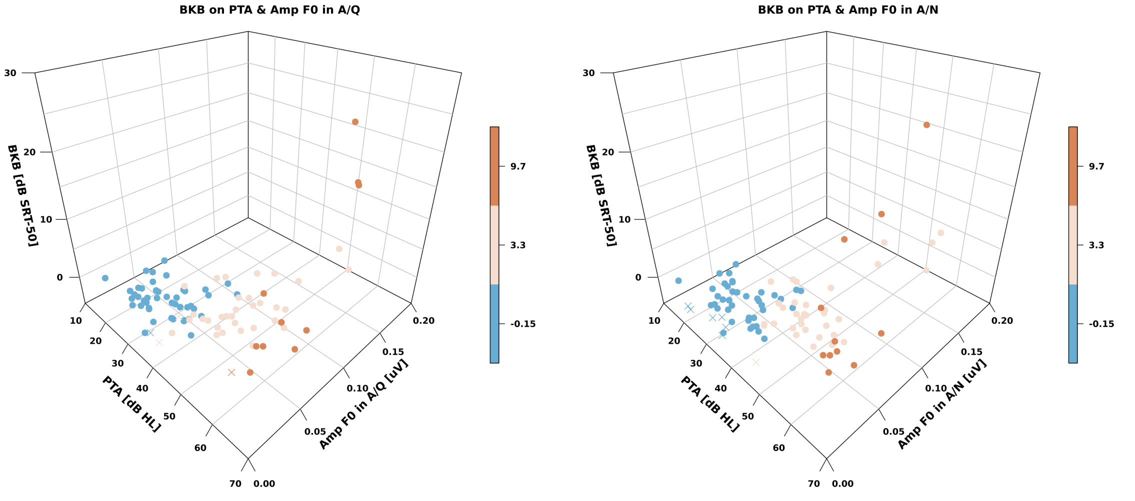

Figure 9: F0 encoding tree. BKB-SIN SRT-50 on PTA & Amp F0 in A/Q (left panel); BKB SRT50 on PTA & Amp F0 in A/N (right panel). Amp: amplitude; A/Q: aided speech-ABRs in quiet; A/N: aided speech-ABRs in noise. Missing F0 encoding are indicated with crosses at 0 uV for graphical convenience.

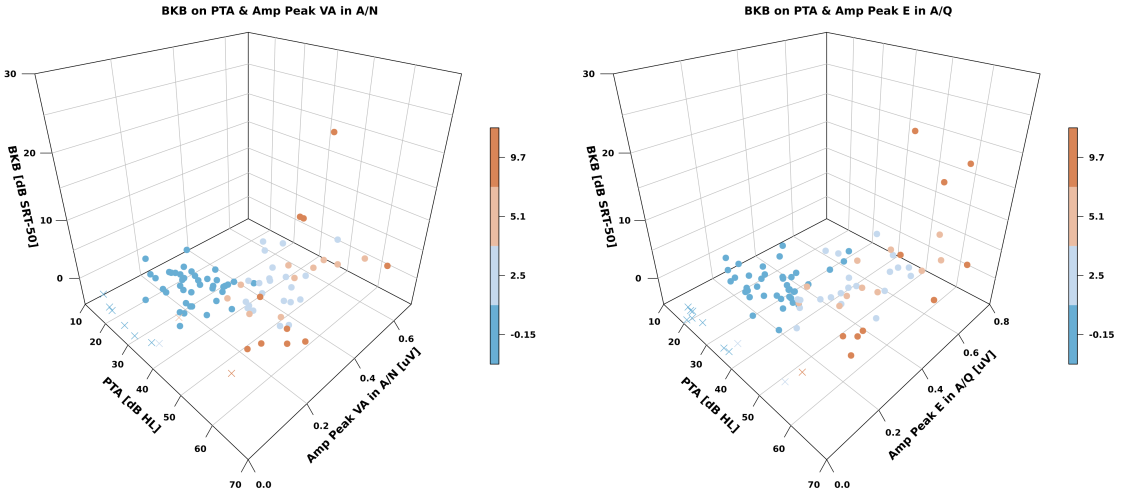

Figure 10: Speech-ABR peaks tree. BKB-SIN SRT-50 on PTA & Amp Peak VA in A/N (left panel); BKB SRT50 on PTA & Amp Peak E in A/Q (right panel). Amp: amplitude; A/N: aided speech-ABRs in noise; A/Q: aided speech-ABRs in quiet. Missing peaks are indicated with crosses at 0 uV for graphical convenience. Note: the y-axis scale is not equal across panels.

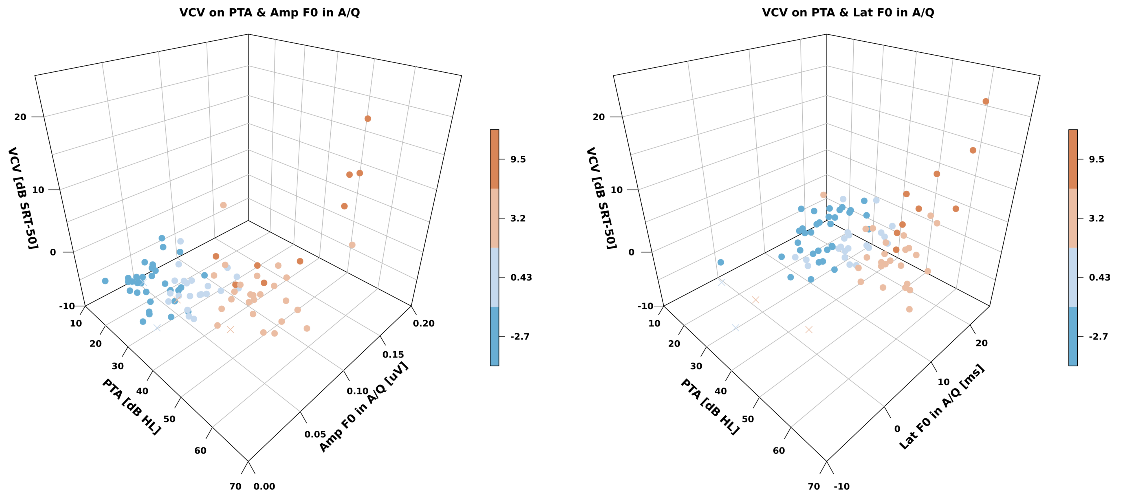

Figure 11: F0 encoding tree. VCV SRT50 on PTA & Amp F0 in A/Q (left panel); VCV SRT50 on PTA & Lat F0 in A/Q (right panel). Amp: amplitude; Lat: latency; A/Q: aided speech-ABRs in quiet. Missing F0 encoding are indicated with crosses at 0 uV/ms for graphical convenience.

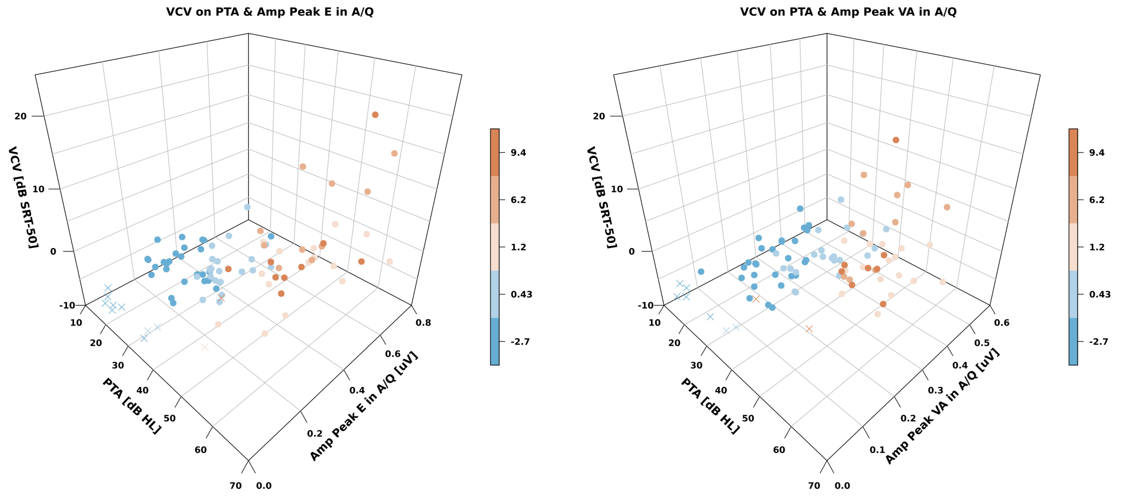

Figure 12: Speech-ABR peaks tree. VCV SRT50 on PTA & Amp Peak E in A/Q (left panel); VCV SRT50 on PTA & Amp Peak F in A/N (right panel). Amp: amplitude; A/Q: aided speech-ABRs in quiet. Missing peaks are indicated with crosses at 0 uV for graphical convenience. Note: the y-axis scale is not equal across panels.

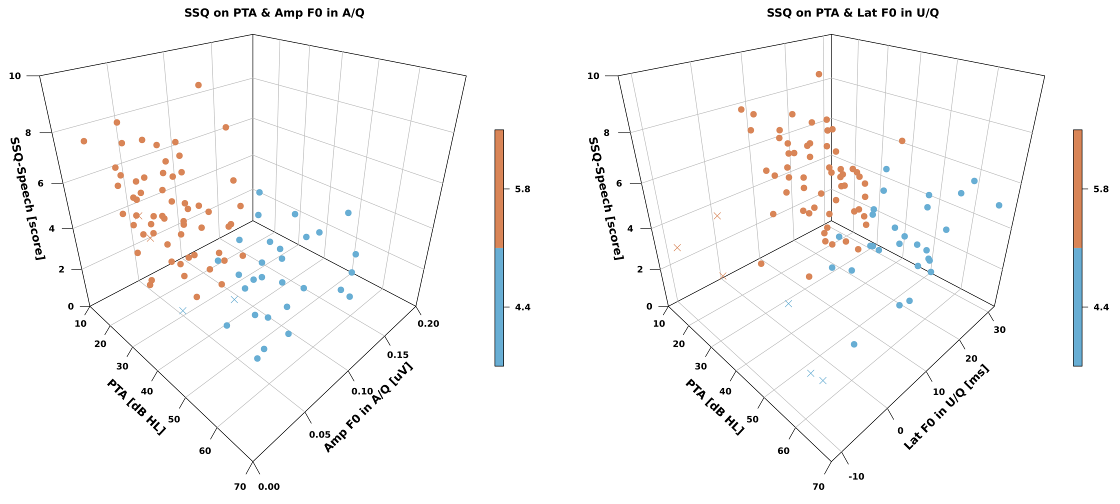

Figure 13: F0 encoding tree. SSQ-Speech score on PTA & Amp F0 in A/Q (left panel); SSQ-Speech score on PTA & Lat F0 in U/Q (right panel). Lat: latency; Amp: amplitude; A/Q: aided speech-ABRs in quiet; U/Q: unaided speech-ABRs in quiet. Missing F0 encoding are indicated with crosses at 0 uV/ms for graphical convenience.

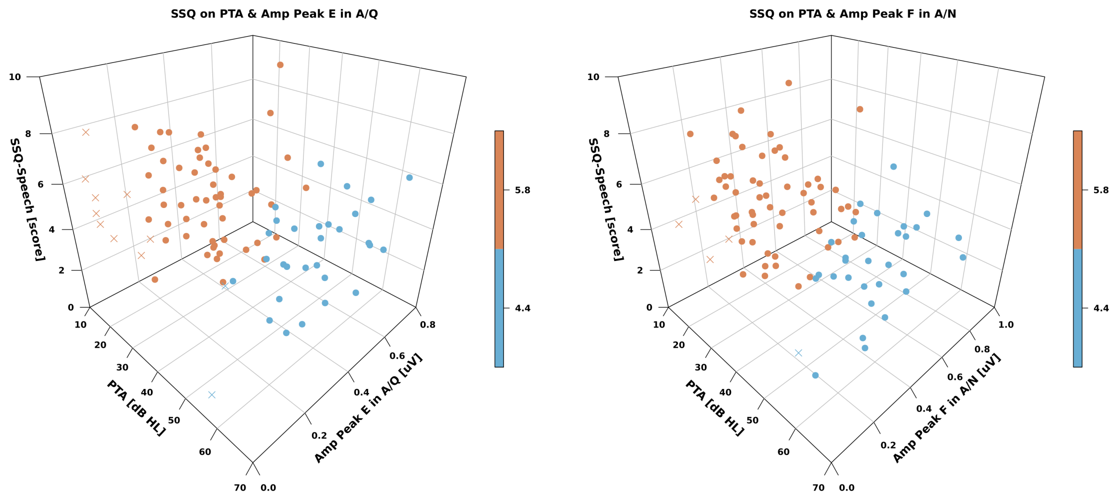

Figure 14: Speech-ABR peaks tree. SSQ-Speech score on PTA & Amp Peak E in A/Q (left panel); SSQ-Speech score on PTA & Amp Peak F in A/N (right panel). Amp: amplitude; A/Q: aided speech-ABRs in quiet; A/N: aided speech-ABRs in noise. Missing peaks are indicated with crosses at 0 uV for graphical convenience. Note: the y-axis scale is not equal across panels.

Table 1: Regression trees summary table. DV: dependent variable; CP: complexity parameter; PRESS: predicted residual error sum of squares statistic; F0: tree using F0 encoding measures; ABR: tree using speech-ABR peaks; Lat: latency; Amp: amplitude; A/Q: aided speech-ABRs in quiet.

DV	Tree	CP	Num Split	Var Split	Relative Error ($1\text{-}R^2$)	Cross-Validation Error (PRESS)
BKB SRT50	F0	0.319	0		1	1.023
BKB SRT50	F0	0.111	1	PTA	0.681	0.875
BKB SRT50	F0	0.016	2	PTA	0.570	0.774
BKB SRT50	ABR	0.319	0		1	1.023
BKB SRT50	ABR	0.111	1	PTA	0.681	0.875
BKB SRT50	ABR	0.024	2	PTA	0.570	0.800
BKB SRT50	ABR	0.020	3	Lat Peak F in A/Q	0.547	0.780
VCV SRT50	F0	0.333	0		1	1.016
VCV SRT50	F0	0.109	1	PTA	0.667	0.836
VCV SRT50	F0	0.053	2	PTA	0.558	0.768
VCV SRT50	F0	0.048	3	Lat F0 in A/Q	0.506	0.747
VCV SRT50	ABR	0.333	0		1	1.016
VCV SRT50	ABR	0.121	1	PTA	0.667	0.836
VCV SRT50	ABR	0.060	2	PTA	0.546	0.788
VCV SRT50	ABR	0.053	3	Amp Peak VA in A/Q	0.485	0.809
VCV SRT50	ABR	0.027	4	Age	0.433	0.771
SSQ Speech	F0	0.165	0		1	1.024
SSQ Speech	F0	0.069	1	PTA	0.835	0.928
SSQ Speech	ABR	0.165	0		1	1.024
SSQ Speech	ABR	0.071	1	PTA	0.835	0.928

Table 2: The three most important variables for each tree. Values are in % in proportion of all variables. Lat: latency; Amp: amplitude; A/Q: aided speech-ABRs in quiet; A/N: aided speech-ABRs in noise; U/Q: unaided speech-ABRs in quiet.

F0 tree		ABR tree	
BKB SRT50			
PTA	82	PTA	64
Amp F0 in A/Q	8	Amp Peak VA in A/N	6
Amp F0 in A/N	6	Amp Peak E in A/Q	5
VCV SRT50			
PTA	43	PTA	41
Amp F0 in A/Q	20	Amp Peak VA in A/Q	11
Lat F0 in A/Q	13	Amp Peak E in A/Q	9
SSQ Speech			
PTA	63	PTA	84
Amp F0 in A/Q	20	Amp Peak E in A/Q	5
Lat F0 in U/Q	8	Amp Peak F in A/N	5
