# Supplementary material for: On prediction of aided behavioural measures using speech auditory brainstem responses and decision trees
Source: PLoS One. 2021 Nov 16;16(11):e0260090. doi: 10.1371/journal.pone.0260090 (PMC8594837; doi:10.1371/journal.pone.0260090)
Supplement: Attachment — Submitted filename: Response to Reviewers.docx [file pone.0260090.s002.docx]

We thank the academic editor and the reviewer for their comments. In our revised manuscript we did our best to address all points raised.

To make it easy for the reviewer to see how we have responded to their comments, we have separated and numbered their comments. We have used italicised font and blue for the reviewer’s comments.

**Reviewer #2:**

 I was pleased to read your article. However, I suggest the following.

Thank you for your comments and suggestions that have helped us improve the quality of the manuscript.

Detailed comments:

1. In participants (line 160), I would advise you to write all participants characteristics instead of asking the reader to find the referred article.

- We have added the following participant information (lines 177 - 181):

“The included participants had acquired bilateral SNHL not exceeding 70 dB HL at low to mid frequencies (i.e., between 250 and 2000 Hz) in the better ear or in the aided ear; and they used at least one HA for 3 months minimum. The participants had no history of learning difficulties, neurological disorders, or cognitive impairments.”

2. In hearing evaluation and HA fitting, it was said otoscopy tympanometry and PTA was performed. But you only explained how PTA was performed. Also, speech audiometry was performed?

- We have added the following information to address otoscopy and tympanometry (lines 187 – 189):

“Ears were examined following the BSA recommended procedure [24]; and tympanometry was performed using the Interacoustics Titan device with a 226-Hz probe tone [25].”

- The new references:

24.	British Society of Audiology. Recommended Procedure: Ear examination. 2016. Available: https://www.thebsa.org.uk/wp-content/uploads/2016/04/OD104-54-Recommended-Procedure-Ear-Examiniation-Sept-2016.pdf

25.	British Society of Audiology. Tympanometry. 2013. Available: https://www.thebsa.org.uk/wp-content/uploads/2013/04/OD104-35-Recommended-Procedure-Tympanometry-.pdf

- Speech audiometry was not performed because the aim of the study was to assess if speech-ABRs predicted aided speech-recognition in noise.

3. How was the hearing status of the participants? So that monoaural fitting was preferred?.

- Regarding the hearing status:

The spreadsheet “BinKhamis_et_al_behavioral_data .xlsx” at <http://doi.org/10.5281/zenodo.2652864> shows test ear PTA (average at 500, 1000, 2000, and 4000 Hz) for each participant.

- In order to show the overall hearing status of the participants, we changed the Fig 1, adding a second panel with nontest-ear pure tone thresholds:

- New caption:

Test-ear (left panel, partially reproduced from [6]) and nontest-ear (right panel) mean (±1 SD) pure tone thresholds (black) with pure tone thresholds for each of the 92 participants (grey).

In the previous caption we wrote wrongly 98 participants, instead of the correct 92. Now corrected, apologies for the error.

- Regarding the monoaural fitting, we have added the following (lines 199 - 201):

Each participant was fitted to NAL-NL2 targets [27] with one Oticon opn1 miniRITE (Oticon A/S, Copenhagen, Denmark) HA “on the better ear, on the aided ear for participants that were monaural hearing aid users, or on the right ear for participants with symmetrical hearing loss that were binaural hearing aid users.”

- Our rationale for fitting one HA is explained in lines 202-207:

Monaural fitting was chosen to avoid the confound of the better-hearing ear driving the response in cases of asymmetrical hearing losses, to evaluate the potential application of speech-ABRs as an outcome measure for individual HA fitting, and to account for test-ear pure tone thresholds in the analyses.

4. In line 183, again study 6 was referred to read to understand the present article

- Because HA fitting and Verification are specific clinical procedures that may be difficult to digest for some readers, especially that the audience for this manuscript are more likely non-clinical, we have added a simplified sentence on the fitting and verification and refer the reader to the original for more details (lines 205-208):

“Each HA was fit using an appropriate receiver per-participant hearing thresholds and fitting was adjusted and verified to meet NAL-NL2 targets [27] according to the BSA recommendations [28]. More details on the implementations of the HA Fitting and Verification are available in [6]”

- The new reference:

28.	British Society of Audiology. Guidance on the verification of hearing devices using probe microphone measurements. 2018. Available: https://www.thebsa.org.uk/wp-content/uploads/2018/05/REMS-2018.pdf

5. In line 190 and line 196, what was the initial background babble?

- Thank you for pointing this out, we have added this (line 218):

“with the initial SNR set at + 6 dB”

- And (lines 224-225):

“, with the initial SNR of +6 dB”

6. In line 236, I would advise you to write the acquisition parameters instead of asking the reader to find the referred article.

- The line “Speech-ABR recording procedure is described in detail in [6].” is removed as we think that the sections “Equipment and recording parameters” and “Stimuli and recording procedure” are exhaustive to replicate the study. We appreciate the sentence was confusing.

7. Why the loudspeaker was placer at 00 azimuth, at 1,3 m for the speech-in-noise test and 450 azimuth for speech-ABR, at 1,1 m?

- This was clarified as follows (lines 271-275):

The different loudspeaker angle between the SIN tests and Speech-ABRs (0° vs. 45° azimuth) was due to the different position of the participants (seated upright vs. supine position), and that the loudspeaker could not be mounted from the ceiling. The different angles were unlikely to have affected the results.

8. In line 427, you say “several studies” but only refer to two studies. The same in line 434.

- Line 478 (ex 427) changed:

“Two”

- In line 485 (ex 434), we prefer to keep using the phrase “Others did not find the association between speech-ABRs and SIN test” because Mamo et al. (2019) did not find this association, while Coffey et al. (2017) found the association using MEG but not using EEG. So, saying “Two” studies did not find an association, would be incorrect.

9. Conclusion, only at line 533 you start your conclusion.

- Yes, because we wanted to summarise our hypothesis, and highlighted key aspects of the study (regression trees and large dataset). We chose to write it this way in order for the reader to get an overall summary along with the conclusion without needing to go back to the manuscript.
