# Supplementary material for: On prediction of aided behavioural measures using speech auditory brainstem responses and decision trees
Source: PLoS One. 2021 Nov 16;16(11):e0260090. doi: 10.1371/journal.pone.0260090 (PMC8594837; doi:10.1371/journal.pone.0260090)
Supplement: Peer review history

# Peer review: Decision Letter 0

**DOI:** 10.1371/journal.pone.0260090.r001

**Authors:** Pedro de Lemos Menezes

21 Sep 2021

PONE-D-21-18372On prediction of aided behavioural measures using Speech Auditory Brainstem Responses and Decision TreesPLOS ONE

Dear Dr. Perugia,

Thank you for submitting your manuscript to PLOS ONE. After careful consideration, we feel that it has merit but does not fully meet PLOS ONE’s publication criteria as it currently stands. Therefore, we invite you to submit a revised version of the manuscript that addresses the points raised during the review process.

As can be seen, the reviewers only suggested minor modifications (reviewer 2). We await the corrected version of the article.

Please submit your revised manuscript by Nov 05 2021 11:59PM. If you will need more time than this to complete your revisions, please reply to this message or contact the journal office at plosone@plos.org. When you're ready to submit your revision, log on to https://www.editorialmanager.com/pone/ and select the 'Submissions Needing Revision' folder to locate your manuscript file.

Please include the following items when submitting your revised manuscript:

- A rebuttal letter that responds to each point raised by the academic editor and reviewer(s). You should upload this letter as a separate file labeled 'Response to Reviewers'.
- A marked-up copy of your manuscript that highlights changes made to the original version. You should upload this as a separate file labeled 'Revised Manuscript with Track Changes'.
- An unmarked version of your revised paper without tracked changes. You should upload this as a separate file labeled 'Manuscript'.

If you would like to make changes to your financial disclosure, please include your updated statement in your cover letter. Guidelines for resubmitting your figure files are available below the reviewer comments at the end of this letter.

If applicable, we recommend that you deposit your laboratory protocols in protocols.io to enhance the reproducibility of your results. Protocols.io assigns your protocol its own identifier (DOI) so that it can be cited independently in the future. For instructions see: https://journals.plos.org/plosone/s/submission-guidelines#loc-laboratory-protocols. Additionally, PLOS ONE offers an option for publishing peer-reviewed Lab Protocol articles, which describe protocols hosted on protocols.io. Read more information on sharing protocols at https://plos.org/protocols?utm_medium=editorial-email&utm_source=authorletters&utm_campaign=protocols.

We look forward to receiving your revised manuscript.

Kind regards,

Pedro de Lemos Menezes, Ph.D

Academic Editor

PLOS ONE

Journal Requirements:

When submitting your revision, we need you to address these additional requirements.

1.Please ensure that your manuscript meets PLOS ONE's style requirements, including those for file naming. The PLOS ONE style templates can be found at

https://journals.plos.org/plosone/s/file?id=wjVg/PLOSOne_formatting_sample_main_body.pdf and https://journals.plos.org/plosone/s/file?id=ba62/PLOSOne_formatting_sample_title_authors_affiliations.pdf.

2. We noted in your submission details that a portion of your manuscript may have been presented or published elsewhere. [The study is a secondary analysis of data published in BinKhamis et al. (2019)] Please clarify whether this [conference proceeding or publication] was peer-reviewed and formally published. If this work was previously peer-reviewed and published, in the cover letter please provide the reason that this work does not constitute dual publication and should be included in the current manuscript.

3. Please ensure that you refer to Figure 2 in your text as, if accepted, production will need this reference to link the reader to the figure.

4. Please review your reference list to ensure that it is complete and correct. If you have cited papers that have been retracted, please include the rationale for doing so in the manuscript text, or remove these references and replace them with relevant current references. Any changes to the reference list should be mentioned in the rebuttal letter that accompanies your revised manuscript. If you need to cite a retracted article, indicate the article’s retracted status in the References list and also include a citation and full reference for the retraction notice.

[Note: HTML markup is below. Please do not edit.]

Reviewers' comments:

Reviewer's Responses to Questions

**Comments to the Author**

1. Is the manuscript technically sound, and do the data support the conclusions?

The manuscript must describe a technically sound piece of scientific research with data that supports the conclusions. Experiments must have been conducted rigorously, with appropriate controls, replication, and sample sizes. The conclusions must be drawn appropriately based on the data presented. 

Reviewer #1: Yes

Reviewer #2: No

**********

2. Has the statistical analysis been performed appropriately and rigorously? 

Reviewer #1: Yes

Reviewer #2: Yes

**********

3. Have the authors made all data underlying the findings in their manuscript fully available?

The PLOS Data policy requires authors to make all data underlying the findings described in their manuscript fully available without restriction, with rare exception (please refer to the Data Availability Statement in the manuscript PDF file). The data should be provided as part of the manuscript or its supporting information, or deposited to a public repository. For example, in addition to summary statistics, the data points behind means, medians and variance measures should be available. If there are restrictions on publicly sharing data—e.g. participant privacy or use of data from a third party—those must be specified.

Reviewer #1: Yes

Reviewer #2: Yes

**********

4. Is the manuscript presented in an intelligible fashion and written in standard English?

PLOS ONE does not copyedit accepted manuscripts, so the language in submitted articles must be clear, correct, and unambiguous. Any typographical or grammatical errors should be corrected at revision, so please note any specific errors here.

Reviewer #1: Yes

Reviewer #2: Yes

**********

5. Review Comments to the Author

Please use the space provided to explain your answers to the questions above. You may also include additional comments for the author, including concerns about dual publication, research ethics, or publication ethics. (Please upload your review as an attachment if it exceeds 20,000 characters)

Reviewer #1: This paper investigated the usefulness of speech-ABR in assessing the benefits of hearing aids objectively. This paper does have the novelty, well written, adequate number of subjects, well analyzed and well discussed. I would recommend this paper for publication.

Reviewer #2: I was pleased to read your article. However, I suggest the following:

1. In participants (line 160), I would advise you to write all participants characteristics instead of asking the reader to find the referred article.

2. In hearing evaluation and HA fitting, it was said otoscopy tympanometry and PTA was performed. But you only explained how PTA was performed. Also, speech audiometry was performed?

3. How was the hearing status of the participants? So that monoaural fitting was preferred?

4. In line 183, again study 6 was referred to read to understand the present article

5. In line 190 and line 196, what was the initial background babble?

6. In line 236, I would advise you to write the acquisition parameters instead of asking the reader to find the referred article.

7. Why the loudspeaker was placer at 00 azimuth, at 1,3 m for the speech-in-noise test and 450 azimuth for speech-ABR, at 1,1 m?

8. In line 427, you say “several studies” but only refer to two studies. The same in line 434.

9. Conclusion, only at line 533 you start your conclusion.

**********

6. PLOS authors have the option to publish the peer review history of their article (what does this mean?). If published, this will include your full peer review and any attached files.

If you choose “no”, your identity will remain anonymous but your review may still be made public.

**Do you want your identity to be public for this peer review?** For information about this choice, including consent withdrawal, please see our Privacy Policy.

Reviewer #1: **Yes: **Mohd Normani Zakaria

Reviewer #2: No

[NOTE: If reviewer comments were submitted as an attachment file, they will be attached to this email and accessible via the submission site. Please log into your account, locate the manuscript record, and check for the action link "View Attachments". If this link does not appear, there are no attachment files.]

While revising your submission, please upload your figure files to the Preflight Analysis and Conversion Engine (PACE) digital diagnostic tool, https://pacev2.apexcovantage.com/. PACE helps ensure that figures meet PLOS requirements. To use PACE, you must first register as a user. Registration is free. Then, login and navigate to the UPLOAD tab, where you will find detailed instructions on how to use the tool. If you encounter any issues or have any questions when using PACE, please email PLOS at figures@plos.org. Please note that Supporting Information files do not need this step.

# Author comment: Author response to Decision Letter 0

**DOI:** 10.1371/journal.pone.0260090.r002

13 Oct 2021

We thank the academic editor and the reviewer for their comments. In our revised manuscript we did our best to address all points raised.

Reviewer #2: 

 I was pleased to read your article. However, I suggest the following.

Thank you for your comments and suggestions that have helped us improve the quality of the manuscript.

Detailed comments:

 1. In participants (line 160), I would advise you to write all participants characteristics instead of asking the reader to find the referred article.

 • We have added the following participant information (lines 177 - 181): 

“The included participants had acquired bilateral SNHL not exceeding 70 dB HL at low to mid frequencies (i.e., between 250 and 2000 Hz) in the better ear or in the aided ear; and they used at least one HA for 3 months minimum. The participants had no history of learning difficulties, neurological disorders, or cognitive impairments.”

 2. In hearing evaluation and HA fitting, it was said otoscopy tympanometry and PTA was performed. But you only explained how PTA was performed. Also, speech audiometry was performed?

 • We have added the following information to address otoscopy and tympanometry (lines 187 – 189):

“Ears were examined following the BSA recommended procedure [24]; and tympanometry was performed using the Interacoustics Titan device with a 226-Hz probe tone [25].”

 • The new references: 

24. British Society of Audiology. Recommended Procedure: Ear examination. 2016. Available: https://www.thebsa.org.uk/wp-content/uploads/2016/04/OD104-54-Recommended-Procedure-Ear-Examiniation-Sept-2016.pdf

25. British Society of Audiology. Tympanometry. 2013. Available: https://www.thebsa.org.uk/wp-content/uploads/2013/04/OD104-35-Recommended-Procedure-Tympanometry-.pdf

 • Speech audiometry was not performed because the aim of the study was to assess if speech-ABRs predicted aided speech-recognition in noise.

 3. How was the hearing status of the participants? So that monoaural fitting was preferred?.

 • Regarding the hearing status: 

The spreadsheet “BinKhamis_et_al_behavioral_data .xlsx” at http://doi.org/10.5281/zenodo.2652864 shows test ear PTA (average at 500, 1000, 2000, and 4000 Hz) for each participant.

 • In order to show the overall hearing status of the participants, we changed the Fig 1, adding a second panel with nontest-ear pure tone thresholds:

 • New caption: 

Test-ear (left panel, partially reproduced from [6]) and nontest-ear (right panel) mean (±1 SD) pure tone thresholds (black) with pure tone thresholds for each of the 92 participants (grey).

In the previous caption we wrote wrongly 98 participants, instead of the correct 92. Now corrected, apologies for the error.

 • Regarding the monoaural fitting, we have added the following (lines 199 - 201):

Each participant was fitted to NAL-NL2 targets [27] with one Oticon opn1 miniRITE (Oticon A/S, Copenhagen, Denmark) HA “on the better ear, on the aided ear for participants that were monaural hearing aid users, or on the right ear for participants with symmetrical hearing loss that were binaural hearing aid users.”

 • Our rationale for fitting one HA is explained in lines 202-207:

Monaural fitting was chosen to avoid the confound of the better-hearing ear driving the response in cases of asymmetrical hearing losses, to evaluate the potential application of speech-ABRs as an outcome measure for individual HA fitting, and to account for test-ear pure tone thresholds in the analyses.

 4. In line 183, again study 6 was referred to read to understand the present article

 • Because HA fitting and Verification are specific clinical procedures that may be difficult to digest for some readers, especially that the audience for this manuscript are more likely non-clinical, we have added a simplified sentence on the fitting and verification and refer the reader to the original for more details (lines 205-208):

“Each HA was fit using an appropriate receiver per-participant hearing thresholds and fitting was adjusted and verified to meet NAL-NL2 targets [27] according to the BSA recommendations [28]. More details on the implementations of the HA Fitting and Verification are available in [6]” 

 • The new reference: 

28. British Society of Audiology. Guidance on the verification of hearing devices using probe microphone measurements. 2018. Available: https://www.thebsa.org.uk/wp-content/uploads/2018/05/REMS-2018.pdf

 5. In line 190 and line 196, what was the initial background babble?

 • Thank you for pointing this out, we have added this (line 218): 

“with the initial SNR set at + 6 dB”

 • And (lines 224-225): 

“, with the initial SNR of +6 dB”

 6. In line 236, I would advise you to write the acquisition parameters instead of asking the reader to find the referred article.

 • The line “Speech-ABR recording procedure is described in detail in [6].” is removed as we think that the sections “Equipment and recording parameters” and “Stimuli and recording procedure” are exhaustive to replicate the study. We appreciate the sentence was confusing.

 7. Why the loudspeaker was placer at 00 azimuth, at 1,3 m for the speech-in-noise test and 450 azimuth for speech-ABR, at 1,1 m?

 • This was clarified as follows (lines 271-275): 

The different loudspeaker angle between the SIN tests and Speech-ABRs (0° vs. 45° azimuth) was due to the different position of the participants (seated upright vs. supine position), and that the loudspeaker could not be mounted from the ceiling. The different angles were unlikely to have affected the results.

 8. In line 427, you say “several studies” but only refer to two studies. The same in line 434.

 • Line 478 (ex 427) changed: 

“Two” 

 • In line 485 (ex 434), we prefer to keep using the phrase “Others did not find the association between speech-ABRs and SIN test” because Mamo et al. (2019) did not find this association, while Coffey et al. (2017) found the association using MEG but not using EEG. So, saying “Two” studies did not find an association, would be incorrect.

 9. Conclusion, only at line 533 you start your conclusion.

 • Yes, because we wanted to summarise our hypothesis, and highlighted key aspects of the study (regression trees and large dataset). We chose to write it this way in order for the reader to get an overall summary along with the conclusion without needing to go back to the manuscript.

Regarding the Journal Requirements: 

(1) The manuscript now meets PLOS ONE's style and file naming requirements. Furthermore, the file formats are now in DOCX (they were in ODT in the previous submission).

(2) The study by BinKhamis et al. (2019) was peer-reviewed and formally published in Trends in Hearing. We appreciate your concern about dual or redundant publication. Considering the COPE’s definition a of redundant publication (https://publicationethics.org/category/keywords/redundant-publication), we have been transparent to acknowledge BinKhamis et al. (2019), and we attached it in the original submission. BinKhamis et al. (2019) investigated 1) the effects of aiding and background noise on speech-ABRs; and 2) the prediction of the aided speech-ABR F0 encoding amplitude and latency on behavioural measures. Here we included also unaided speech-ABR F0 encoding and aided and unaided speech-ABR peaks in the analysis. Some hypotheses of the current study (i.e., non-linearity and sub-groups) were built upon the results obtained by BinKhamis et al. (2019). Furthermore, we used the regression tree to model the data, which is a new, different and non-linear approach, and as such it merits to be described in a separate paper. To the best of our knowledge, this is the first study that used regression trees to predict behavioural measures using speech-ABRs, thus the novelty and difference of our approach to BinKhamis et al. (2019) is strong. The current study is aimed at a different reader group than BinKhamis et al. (2019). However, BinKhamis et al. (2019) and the current study overlap in some parts of the materials and methods section, we believe this minor overlap is legitimate.

(3) Reference to Figure 2 is now in the text.

(4) We prepared the reference list using a reference management software (Zotero), so we believe that it is complete and correct. We used the Retraction Watch Database to avoid citing retraced papers. In order to address the reviewer’s comments, we added three references.

Attachment Submitted filename: Response to Reviewers.docx Click here for additional data file.

# Editor report: Decision Letter 1

**DOI:** 10.1371/journal.pone.0260090.r003

**Authors:** Pedro de Lemos Menezes

3 Nov 2021

On prediction of aided behavioural measures using Speech Auditory Brainstem Responses and Decision Trees

PONE-D-21-18372R1

Dear Dr. Perugia,

We’re pleased to inform you that your manuscript has been judged scientifically suitable for publication and will be formally accepted for publication once it meets all outstanding technical requirements.

Within one week, you’ll receive an e-mail detailing the required amendments. When these have been addressed, you’ll receive a formal acceptance letter and your manuscript will be scheduled for publication.

An invoice for payment will follow shortly after the formal acceptance. To ensure an efficient process, please log into Editorial Manager at http://www.editorialmanager.com/pone/, click the 'Update My Information' link at the top of the page, and double check that your user information is up-to-date. If you have any billing related questions, please contact our Author Billing department directly at authorbilling@plos.org.

If your institution or institutions have a press office, please notify them about your upcoming paper to help maximize its impact. If they’ll be preparing press materials, please inform our press team as soon as possible -- no later than 48 hours after receiving the formal acceptance. Your manuscript will remain under strict press embargo until 2 pm Eastern Time on the date of publication. For more information, please contact onepress@plos.org.

Kind regards,

Pedro de Lemos Menezes, Ph.D

Academic Editor

PLOS ONE

Additional Editor Comments (optional):

Reviewers' comments:

# Editor report: Acceptance letter

**DOI:** 10.1371/journal.pone.0260090.r004

**Authors:** Pedro de Lemos Menezes

5 Nov 2021

PONE-D-21-18372R1 

On prediction of aided behavioural measures using Speech Auditory Brainstem Responses and Decision Trees 

Dear Dr. Perugia:

I'm pleased to inform you that your manuscript has been deemed suitable for publication in PLOS ONE. Congratulations! Your manuscript is now with our production department. 

If your institution or institutions have a press office, please let them know about your upcoming paper now to help maximize its impact. If they'll be preparing press materials, please inform our press team within the next 48 hours. Your manuscript will remain under strict press embargo until 2 pm Eastern Time on the date of publication. For more information please contact onepress@plos.org.

If we can help with anything else, please email us at plosone@plos.org. 

Thank you for submitting your work to PLOS ONE and supporting open access. 

Kind regards, 

PLOS ONE Editorial Office Staff

on behalf of

Dr. Pedro de Lemos Menezes 

Academic Editor

PLOS ONE
